# A Rationale-Conditioned Image-Contrast Audit of OCT Dependence in Vision-Language Models for Anti-VEGF Treatment-Response Prediction

**DOI:** 10.3390/bioengineering13080912

**Published:** 2026-08-12

**Authors:** Wonbong Jang, Gwon Yul Jo, Siyun Lee, Shin Jeong Yoon, Gi Young Lee, Hee-Eun Lee, Tae Hyung Kim, Jong Won Baek, Joonhyung Kim

**Affiliations:** 1 Clear Eye Clinic, 181-26 Jijedongsak 2-ro, Pyeongtaek 18029, Republic of Korea; xhdtlswk7@gmail.com; 2 BioNexus Inc., Gwanggyo Business Center, 156 Gwanggyo-ro, Yeongtong-gu, Suwon 16506, Republic of Korea; gwonyul.jo@bionexus.kr (G.Y.J.);; 3 Department of Ophthalmology, CHA Bundang Medical Center, CHA University School of Medicine, 59 Yatap-ro, Bundang-gu, Seongnam 13496, Republic of Korea

**Keywords:** vision-language models, optical coherence tomography, anti-VEGF, treatment-response prediction, generated rationale, direct image contrast, clinical AI evaluation, distribution shift, multimodal fusion

## Abstract

Vision-language models (VLMs) are increasingly evaluated for retinal image interpretation, but end-task discrimination does not establish whether a prediction depends on the optical coherence tomography (OCT) image. We evaluated RetinaVLM, LLaVA-Med, and Qwen3.6-27B under zero-shot and parameter-efficient adaptation using an APTOS-2021 cohort (128 training, 21 validation, and 69 test eyes) and a 100-eye cross-site stress-test cohort. The primary readout compared forced-choice continue/stop scores obtained with a real OCT B-scan and a uniform-grey image while holding the generated rationale fixed; it therefore estimates a rationale-conditioned direct image contrast rather than total image dependence. Across 22 internal cells, 21 confidence intervals included an AUC of 0.5, while one Qwen zero-shot cell was inversely aligned (AUC 0.354, 95% CI 0.224–0.493). No positively aligned cells survived the Benjamini–Hochberg adjustment. Selected backbones produced image-responsive biomarker outputs, including pigment epithelial detachment balanced accuracy up to 0.93, whereas a five-field tabular reference model achieved decision AUC 0.731 (95% CI 0.603–0.846). Similar direct-contrast findings occurred in the second-site stress test. Because the generated rationales were not verified as faithful representations of latent computation, the grey image was out of distribution, and the cohorts were small, null direct contrasts cannot show that the models ignored OCT or establish equivalence to no discrimination.

## 1. Introduction

Anti-vascular endothelial growth factor (anti-VEGF) intravitreal therapy is standard care for neovascular age-related macular degeneration (nAMD) and diabetic macular edema (DME), and treatment is commonly adjusted over repeated visits using serial OCT, visual acuity, treatment interval, prior response, and other clinical factors [1,2,3]. The present study does not model this full clinical pathway; it evaluates a dataset-provided binary continue/stop label as a retrospective benchmark. Baseline and follow-up OCT biomarkers carry prognostic information about subsequent outcomes [4,5,6], age-related macular degeneration imposes a large global burden [7], and deep networks can detect or segment retinal fluid [8,9] and predict treatment-related outcomes from baseline OCT [10,11,12]. Retinally specialized, biomedical-generalist, knowledge-augmented, and general-purpose VLMs provide candidate platforms for image-plus-text reasoning [13,14,15,16,17,18]. Multimodal retinal AI studies in other diseases also show that structured clinical or laboratory variables can add complementary information to retinal images: a multicenter chronic kidney disease model combined fundus photographs with urine dipstick data, and a Parkinson disease model combined fundus photographs with demographic features while showing lower performance under external testing [19,20]. These studies motivate explicit modality ablation and transportability analysis rather than an assumption that an image-only score represents a clinically complete decision.

Most medical-image AI systems are evaluated primarily by end-task discrimination, typically accuracy or area under the receiver-operating-characteristic curve (AUC), sometimes accompanied by attention or saliency maps. Medical VLM benchmarks have documented hallucination and inconsistency despite acceptable output-level performance [21,22]. A single discrimination statistic can reflect genuine image information, label prevalence, prompt construction, or text carried in a generated rationale. Multimodal foundation models may rely disproportionately on text [23]; generated rationales may not faithfully represent the computation producing an answer [24,25,26,27,28,29]; and forced-choice probabilities are sensitive to surface form, answer order, and prompt calibration [30,31]. Gradient-based attribution, attention visualization, concept probing, and counterfactual explanation address related but different questions and also require careful validation [32,33,34,35,36,37,38]. These concerns motivate complementary intervention-based audits, but no single audit or explanation method constitutes a complete causal test of image use [39,40,41].

Parameter-efficient adaptation makes it practical to compare factual-rationale supervision, attention regularization, and synthetic counterfactual training across heterogeneous backbones [42,43]. Retinal segmentation pipelines provide candidate region masks [8,9], while catastrophic interference and out-of-distribution generalization can alter surface behavior without reliably transferring the intended capability [44,45,46,47]. The present work does not introduce a new causal estimand, statistical test, or debiasing algorithm. Its contribution is an ophthalmology-specific audit configuration that combines rationale-conditioned image replacement, option-order-symmetric scoring, multi-backbone adaptation, separate biomarker and treatment-related readouts, output-coverage reporting, and cross-site stress testing.

We therefore evaluate a narrowly defined methodological question: under the tested prompts, checkpoints, and adaptation settings, does the real OCT image make a correctly aligned direct contribution to the final binary score after the generated rationale is held fixed? The analysis uses one pre-treatment OCT B-scan, dataset-provided continue/stop and responder labels, the APTOS-2021 development cohort (69 held-out test eyes), and a heterogeneous 100-eye cross-site stress-test cohort across three VLM backbones [12,13,14,18]. This estimand is narrower than total image dependence because it conditions on a potential mediator. We do not claim that the contrast captures the image-to-rationale-to-answer pathway, validates the generated rationale as a faithful account of latent computation, establishes an information-theoretic ceiling, or represents a universal retina-specialist treatment recommendation:

- F1—A rationale-conditioned direct-image audit. The protocol combines option-order-symmetric forced-choice scoring with a real-image versus uniform-grey single-null log-likelihood contrast and bootstrap confidence intervals. The resulting quantity is interpreted as a controlled direct image contrast conditional on the generated rationale, not as formal pointwise mutual information or a measure of total causal image dependence.
- F2—A multi-backbone adaptation matrix. Zero-shot evaluation, factual-rationale supervised fine-tuning, attention-KL regularization, and synthetic fluid-occlusion training are compared, with selected metadata-prompt variants, to determine how each intervention changes observable perception and decision scores under the tested conditions.
- F3—A qualified perception–decision dissociation. Selected supervised arms produce image-responsive biomarker calls on stronger visual backbones, whereas the reported decision contrasts do not show a consistently positive correctly aligned direct image contribution. This finding does not exclude image effects mediated through the generated rationale.
- F4—Cross-site distribution-shift stress testing. A second-site cohort is used to examine transportability under differences in diagnosis, treatment, prevalence, and acquisition context. It is treated as an exploratory stress test rather than formal external clinical validation. Potential sources of shift are discussed, but they were not independently manipulated, and cell-wise results are not used to claim equivalence to no discrimination.

The practical implication is correspondingly limited: end-task AUC should be accompanied by modality ablation, rationale-aware analysis, output-coverage reporting, and conventional image and tabular comparators. Longitudinal and multimodal inputs are plausible design directions, supported by prior retinal AI studies [5,6,19,20], but the present experiments do not establish that they are necessary or sufficient remedies.

### 1.1. Related Work

#### 1.1.1. Medical Vision-Language Models and Retinal Multimodal Decision Support

Instruction-tuned multimodal assistants such as LLaVA-Med [14] and Med-Flamingo [15] established a general image-plus-prompt interface, and this approach has been specialized to retinal imaging. RetinaVLM uses retinal curricula [13]; general-purpose VLM families such as Qwen can be adapted by prompting or low-rank modules [18]; and knowledge-augmented systems combine visual representations with structured medical knowledge [16,17]. Outside the VLM paradigm, purpose-built convolutional networks predict anti-VEGF-related outcomes or treatment intervals from OCT [3,10,11,12]. Multimodal retinal systems also illustrate the potential and the limitations of adding structured data: Bhak et al. combined fundus images with urine dipstick variables for chronic kidney disease and observed gains over an image-only model, whereas Ahn et al. combined fundus images with demographic features for Parkinson disease severity and reported lower discrimination in external data [19,20]. These studies support explicit comparison of image-only, tabular-only, and fused models and careful separation of internal performance from transportability. Medical VLM benchmarks such as CARES and MedHEval further show that output-level accuracy and consistency do not by themselves establish image dependence [21,22].

#### 1.1.2. Faithfulness, Image-Null Contrasts, and Explanation Evaluation

Generated rationales can be unfaithful to the computation that produced an answer [24,25,26,27,28,29] and forced-choice probabilities can be distorted by surface form, position, and prompt priors [30,31]. Attention, gradient-based attribution, concept-based probing, and counterfactual explanations can localize or manipulate features associated with an output, but none alone establishes causal grounding or clinical validity [32,33,34,35,36,37,38]. The generated rationale in this study is therefore treated as an observed conditioning variable, not as a verified representation of latent computation. Let V_real denote the clinical OCT, V_grey the uniform-grey input, M_real the rationale generated from V_real, and Y(V,M) the terminal score. Comparing Y(V_real,M_real) with Y(V_grey,M_real) estimates a controlled direct image contrast after M_real is fixed. It blocks the mediated pathway V → M(V) → Y and cannot establish the total causal contribution of the image. The proposed audit is complementary to established explanation methods rather than a replacement for them.

#### 1.1.3. Parameter-Efficient Adaptation and Exploratory Grounding Interventions

Instruction tuning and low-rank adaptation can alter model behavior efficiently [42,43]. Guided-attention and explanation-regularized training have been used to encourage models to focus on annotated regions [48,49], while retinal fluid segmentation systems provide candidate masks for such interventions [8,9]. However, attention alignment is not equivalent to causal decision use, and catastrophic interference or limited transfer can arise during adaptation [44,45,46,47]. In this study, the attention-KL and fluid-occlusion arms are therefore treated as exploratory engineering interventions. Their failure to improve the reported direct-effect score is not evidence that attention supervision or counterfactual training is ineffective in general.

#### 1.1.4. Tabular Reference Models and Multimodal Fusion

A separate question is how much predictive information can be extracted from a single cross-sectional visit. Baseline OCT biomarkers have modest prognostic value [4], whereas repeated imaging and early-treatment dynamics can improve prediction [5,6]. Fluid presence alone is also an incomplete treatment signal because SRF may be tolerated and different fluid compartments have different prognostic associations [1,2]. Existing OCT prediction models report image-only or image-plus-metadata discrimination [3,10,11,12]. More broadly, retinal multimodal models have demonstrated that structured clinical or laboratory data can complement fundus images, although the magnitude of benefit and external transportability vary across tasks [19,20]. We therefore use logistic regression on the same five pre-treatment fields as a tabular reference model. It is not an information-theoretic ceiling and does not, by itself, identify the mechanism of any VLM fusion gap.

## 2. Materials and Methods

**Figure 1:**
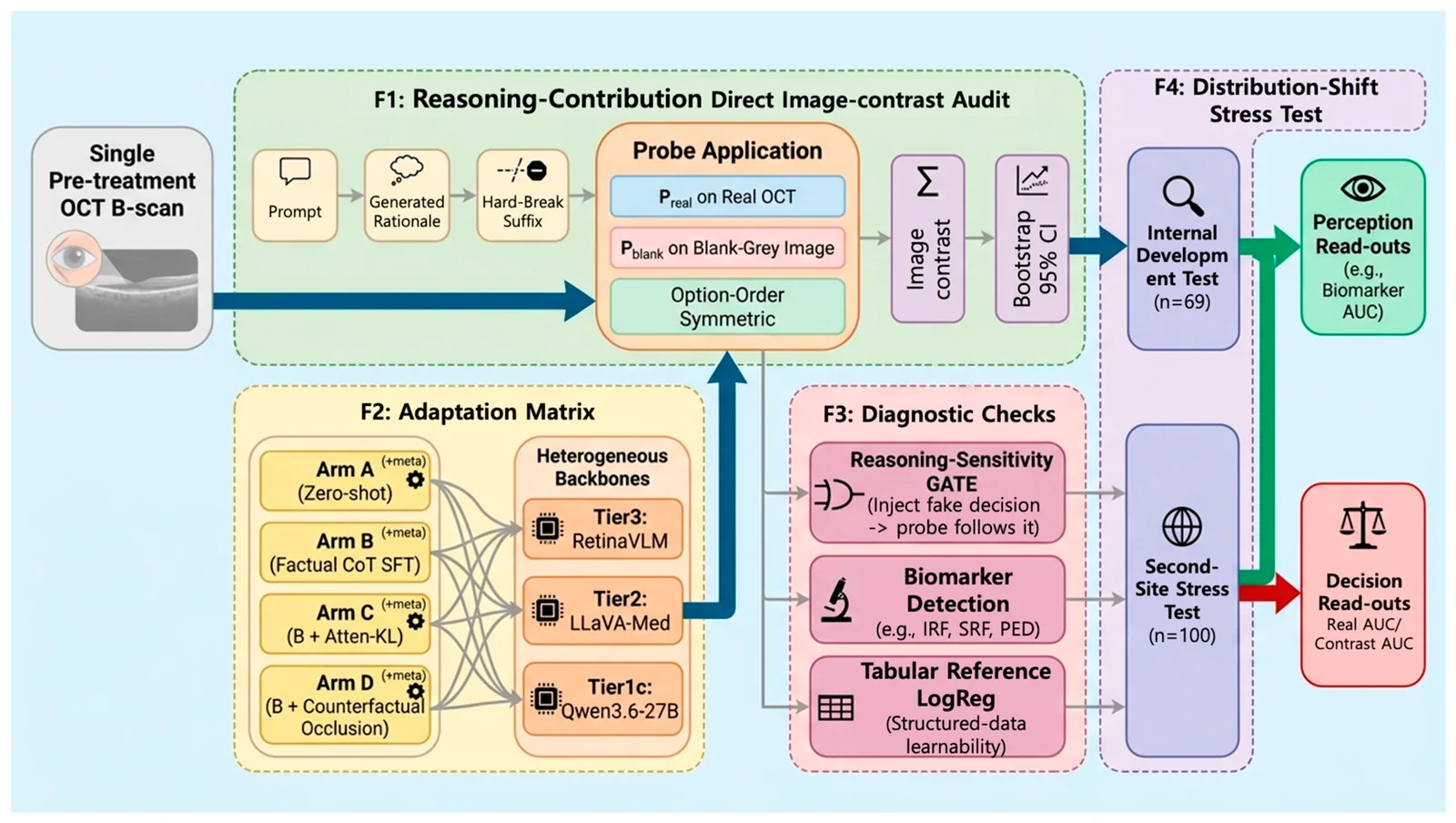
Scope of the audit protocol and adaptation matrix. The real OCT and a uniform-grey image are scored while the model-generated rationale is held fixed. This estimates a controlled direct image contrast conditional on the rationale; the image–rationale–answer pathway is not measured. The diagnostic checks contextualize the readout but do not fully validate visual mediation. The second-site cohort is a distribution-shift stress test, not definitive clinical external validation.

We describe this study as an audit protocol rather than a validated instrument. The following sections define the cohorts and endpoints, model and adaptation conditions, controlled direct image contrast, diagnostic checks, biomarker readouts, and exploratory cross-site analyses. Figure 1 summarizes the scope: the reported contrast addresses the direct image-to-final-score path conditional on a fixed generated rationale and does not quantify image information mediated through that rationale.

### 2.1. Cohorts and Datasets

The internal development cohort is the APTOS-2021 anti-VEGF challenge dataset of paired pre- and post-treatment macular OCT B-scans [12]. The APTOS source images were acquired using the Spectralis OCT platform (Heidelberg Engineering GmbH, Heidelberg, Germany) [12]. Among 218 eyes with available masks, the patient-level split comprised 128 training, 21 validation, and 69 test eyes. The primary endpoint is the dataset-provided binary continue/stop challenge label (test distribution: 44 continue and 25 stop). It was not independently re-adjudicated by masked retina specialists and is analyzed as a retrospective treatment-related benchmark rather than a guideline recommendation or a complete representation of treat-and-extend care. The secondary composite responder label identifies a good response when central subfield thickness decreases by at least 25 μm or when the source-defined visual-acuity improvement variable (ΔVA) is at least 0.1; the OCT4DME source dataset records pre- and post-treatment visual acuity in logMAR [12]. A four-category change-in-CST outcome is used descriptively. Intraretinal fluid (IRF) and subretinal fluid (SRF) are fluid-compartment labels; pigment epithelial detachment (PED) is a structural RPE-elevation biomarker. Five pre-treatment variables—age, sex, planned agent, baseline visual acuity, and baseline central subfield thickness—form the metadata condition; post-treatment and change variables are excluded to prevent target leakage.

All partitions were created at the patient level. When both eyes from one patient were available, both eyes were assigned to the same partition; no bilateral pair crossed the training, validation, and test sets. Adapter optimization was restricted to the 128 training eyes, checkpoint selection to the 21 validation eyes, and the 69 test eyes were excluded from optimization, prompt selection, preprocessing selection, and model selection. Bootstrap resampling used the patient as the clustering unit.

The second-site cohort contains 100 unseen eyes and is used as a cross-site distribution-shift stress test rather than formal external clinical validation. Its endpoint prevalence, biomarker prevalence, diagnoses, treatments, and acquisition context differ substantially from APTOS. The cohort includes DME (26), CNVM (25), PCV (18), BRVO (11), NVE (8), CRVO (4), PDR (3), myopic CNV (3), and CSC (2), with aflibercept (62), faricimab (20), ranibizumab (15), dexamethasone intravitreal implant (Ozurdex; AbbVie Inc., North Chicago, IL, USA) (2), and brolucizumab (1). CNVM is retained as the source-dataset label rather than a harmonized etiologic category. Because Ozurdex is a corticosteroid implant and treatment policies were heterogeneous, the pooled cohort is interpreted as an intravitreal-treatment stress test. Device, disease, regimen, prior-treatment, prevalence, and B-scan-selection differences are plausible contributors to the shift; they were not independently manipulated, so the analysis does not attribute transportability changes to a single source.

### 2.2. Backbones and Adaptation Matrix (F2)

The locked study report identifies three heterogeneous VLM protocol labels: RetinaVLM-Specialist [13], LLaVA-Med-v1.5-Mistral-7B [14], and Qwen3.6-27B [18], with Qwen adapted using 4-bit QLoRA. For reproducibility documentation, these labels were mapped to the public repositories RobbieHolland/RetinaVLM (RetinaVLM-Specialist subfolder), Microsoft/llava-med-v1.5-mistral-7b, and Qwen/Qwen3.6-27B, respectively. The current public revisions inspected during manuscript revision are reported in Supplementary Table S1. However, the original execution environment did not preserve independent weight-file checksums or sufficient provenance to confirm that those public revisions are byte-identical to the historical checkpoints used in the locked run. The models are therefore treated as heterogeneous research comparators, and bit-for-bit checkpoint replication is not claimed.

The adaptation matrix includes zero-shot evaluation (arm A), factually generated-rationale supervised fine-tuning (arm B), arm B plus an attention-KL term toward fluid masks (arm C), and arm B plus synthetic fluid-removal examples (arm D), each with a metadata-prompt variant where applicable. Arm C was unavailable for Qwen, yielding six zero-shot and 16 trained cells (22 total). Arms B and C used factual training rows; arm D used factual plus counterfactual rows. Each trained arm used LoRA rank 16, alpha 32, dropout 0.05, no bias, and target modules q_proj and v_proj; training comprised three epochs with AdamW, learning rate 1 × 10^−4^, and gradient-norm clipping at 1.0. Arm C added a forward-KL term with λ = 0.5 toward a pooled fluid-mask target, and arm D used dilated fluid masks followed by TELEA inpainting. Arm C and arm D are exploratory because the masks lack APTOS pixel-level ground truth, PED is not represented by the fluid mask, and the synthetic “fluid removed → stop” target is not a universal clinical rule. One training realization was used per cell; repeated-seed retraining was not performed.

The common canonical input retained the full pre-treatment OCT B-scan after a fixed crop beginning at x = 520 and resizing to width 720; each backbone then applied its native processor. The supervised target was programmatically rendered from available annotations in four steps: visual findings (IRF/SRF/PED), inferred disease activity, the dataset-provided continue/stop target, and the composite responder target. Metadata prompts prepended age, sex, planned agent, baseline visual acuity, and baseline central subfield thickness. At test time, each model generated a new rationale from the real OCT and, where applicable, only these pre-treatment fields; the ground-truth decision and post-treatment outcomes were not included in the prompt. The exact hard-break scoring suffix, option-order procedure, and all available configuration details are provided in the Supplementary Materials. The analysis pipeline used Python (Python Software Foundation, Wilmington, DE, USA), PyTorch (Meta Platforms, Inc., Menlo Park, CA, USA), Transformers and PEFT (Hugging Face, Inc., New York, NY, USA), bitsandbytes (open-source software), and OpenCV (OpenCV team, open-source software) for TELEA inpainting. Exact historical package versions and hardware identifiers were not preserved in the locked report and were not retrospectively inferred; this limits bit-for-bit environment replication.

### 2.3. Reasoning-Conditioned Image-Null Direct-Effect Audit (F1)

Estimand. Let V_real denote the clinical OCT image, V_grey the uniform-grey input, M_real the generated rationale obtained from V_real, and Y(V,M) the terminal forced-choice probability assigned to “continue.” The primary contrast is D_direct = Y(V_real,M_real) − Y(V_grey,M_real). Because M_real is held fixed, D_direct estimates a rationale-conditioned, controlled direct image contribution and blocks the mediated pathway V → M(V) → Y. A model could encode relevant image information in M_real and subsequently produce its final answer from that text; such a system could show a small D_direct despite substantial end-to-end image dependence. Here, M_real was the model’s own test-time output, not text copied from the supervised target or constructed from the test label.

Operational score. The prompt and generated rationale were followed by the hard-break suffix “=== CLINICAL DECISION === Options: [continue, stop] Final Choice:”. Length-normalized log probabilities for “continue” and “stop” were scored under both option orders and averaged. Each eye therefore required eight scoring passes: two answer orders × two image conditions × two option phrases. The real-image score was compared with the uniform-grey condition. The original analysis files label the contrast and its AUC as PMI and pmiAUC; these labels are retained only where embedded in legacy figures or tables. The quantity is interpreted here as a single-null image-contrast log-likelihood score, not formal pointwise mutual information. Its magnitude is interpreted only together with direction relative to the reference label.

Null-image scope and interpretation. A uniform-grey image is an extreme out-of-distribution intervention that removes low-level OCT statistics as well as patient-specific pathology. The resulting contrast may therefore reflect both the removal of relevant image information and the model’s response to global visual distribution shift. No patient-shuffled OCT, label-matched wrong-patient OCT, blurred, phase-scrambled, patch-shuffled, retinally masked, pathology-inpainted, or true no-image ensemble was available in the locked analysis. Accordingly, the reported score is specific to this single null construction and cannot be generalized to all forms of visual ablation. A 95% CI including AUC 0.5 is described as inconclusive, not equivalent to no discrimination; an interval wholly below 0.5 is reported as inverse alignment.

### 2.4. Diagnostic Checks and Their Scope (F3)

Three diagnostic checks are reported. First, the reasoning-sensitivity GATE verifies that the terminal forced-choice score responds to an injected decision statement; this is a text-sensitivity check and does not validate image-to-rationale mediation. Second, biomarker calls assess whether selected backbones respond to OCT content on a different task; success is label- and backbone-specific and does not guarantee treatment-related grounding. Third, logistic regression on the five pre-treatment fields shows whether the endpoint is learnable from those variables; it is a tabular reference model, not a ceiling. The generated rationale is treated as an observed conditioning variable rather than a verified explanation of latent computation. Together, the checks contextualize the score but do not validate rationale fidelity, the uniform-grey null, or the complete decision pathway.

### 2.5. Responder and Per-Label Biomarker Readouts

Responder and biomarker outputs are evaluated separately from the primary direct-effect estimand. Biomarker performance is reported per label using coverage-penalized balanced accuracy and image-contrast AUC where available; nonanswers are reflected in output coverage. IRF and SRF are fluid compartments, whereas PED is a structural biomarker. A collapse of deterministic predictions under a uniform-grey image indicates image responsiveness, but it does not prove pixel-level localization, exclude image-based shortcuts, or establish faithful causal use of the biomarker. These outputs are therefore interpreted as exploratory task-level checks rather than validated localization measures.

### 2.6. Cross-Site Stress Test and Statistical Analysis (F4)

APTOS-trained adapters were applied without retraining to the 100-eye second-site cohort as a distribution-shift stress test. A five-field logistic model refitted by five-fold out-of-fold estimation within the second site is a local reference model, not external validation of the internally fitted tabular model. AUCs are summarized with patient-clustered bootstrap 95% confidence intervals (5000 replicates; seed 0). Confidence intervals are nominal. Two-sided bootstrap *p*-values relative to AUC 0.5 were adjusted by the Benjamini–Hochberg procedure within the 22-cell decision family and, separately, within the 22-cell responder family. No clinically justified equivalence margin was prespecified; therefore, failure to reject AUC 0.5 is not interpreted as equivalence. Observed post-hoc power was not used because it does not resolve the precision limitation evident in the confidence intervals. The uncertainty reflects test-set resampling but not model-retraining variability because only one training realization was available. Calibration, Brier score, log loss, operating-point metrics, and selective-risk analyses were not available in the locked outputs.

## 3. Results

### 3.1. Internal Zero-Shot Direct-Effect Audit

**Figure 2:**
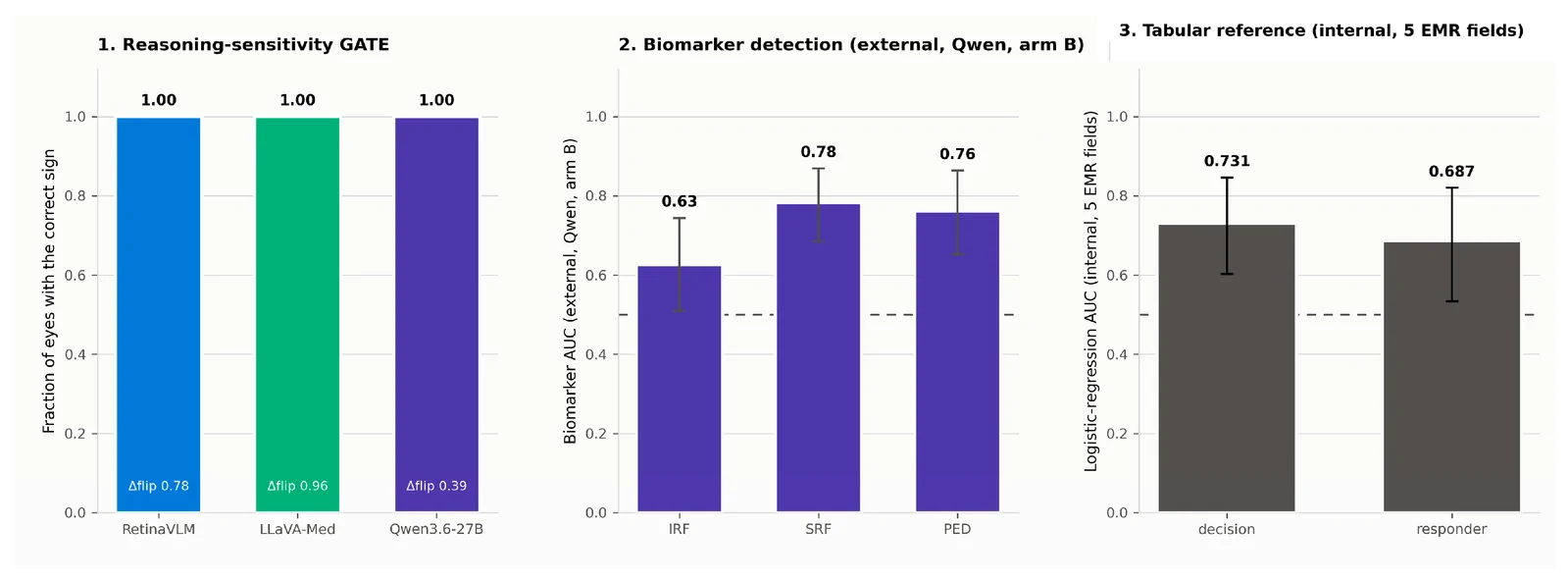
Diagnostic checks and their scope. The reasoning GATE reports the fraction of eyes whose terminal score moved in the injected textual direction (1.00 for all three backbones). The biomarker panel reports external Qwen per-label AUCs (IRF 0.63, SRF 0.78, PED 0.76), and the tabular panel reports internal logistic-regression AUCs (decision 0.731 [0.603, 0.846]; responder 0.687 [0.534, 0.820]). The horizontal dashed line in the biomarker and tabular panels marks AUC = 0.5. These checks demonstrate text sensitivity, selected image-responsive biomarker discrimination, and structured-data learnability, respectively; they do not validate rationale fidelity or total visual mediation.

Across the six zero-shot cells, no correctly aligned direct contrast had a 95% CI wholly above AUC 0.5 (Table 1). Five estimates were inconclusive relative to 0.5, while the Qwen image-only condition was inversely aligned (AUC 0.354, 95% CI 0.224–0.493). Reversing the score direction would yield an AUC of approximately 0.646, indicating systematic misalignment rather than an absence of discriminative information. LLaVA-Med showed very small absolute real-versus-grey score changes, whereas RetinaVLM and Qwen showed larger perturbations that were not consistently aligned with the recorded label. These findings concern the rationale-conditioned direct path only and do not determine whether image information was mediated through the generated rationale. LLaVA-Med generated empty outputs for 17 of 69 A_meta cases; the reported estimate is a complete-case analysis with 52 evaluable cases and 75% output coverage. The three diagnostic checks are summarized in Figure 2.

### 3.2. Reported Trained-Cell Matrix: No Consistently Positive Direct Image Contribution

**Figure 3:**
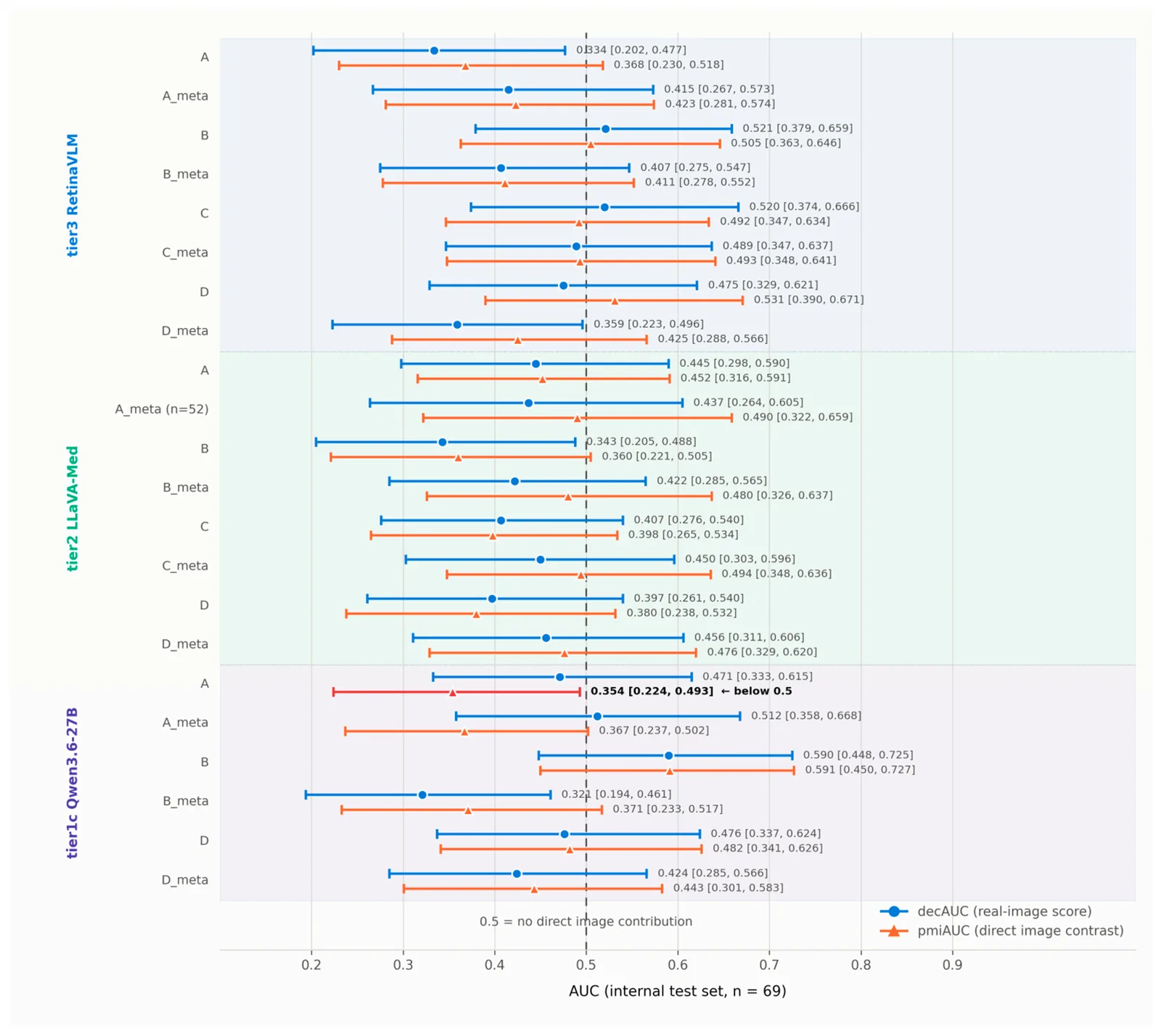
Internal real-image AUCs and rationale-conditioned direct-contrast AUCs for all 22 cells with bootstrap 95% confidence intervals. The embedded panel retains the legacy label pmiAUC. Twenty-one direct-contrast intervals include 0.5; Qwen arm A lies below 0.5 (0.354 [0.224, 0.493]) and is inversely aligned. It is not significant after Benjamini–Hochberg adjustment across the 22-cell decision family (q = 0.37). LLaVA-Med A_meta is reported complete-case (*n* = 52).

Among the 16 trained cells in Table 2, no correctly aligned direct image contrast had a 95% CI wholly above 0.5. The largest positive point estimate was Qwen arm B (0.591, 95% CI 0.450–0.727), which remained imprecise and inconclusive. Several estimates were below 0.5, and Qwen metadata arms showed large perturbations with inverse or weak alignment. Continue recommendations collapsed to 1.00 in multiple B/C conditions, indicating hard-output mode collapse rather than clinically useful discrimination. The six zero-shot cells and 16 trained cells together constitute the complete 22-cell internal design. The results support only the statement that the tested adaptations did not yield a consistently positive direct image contribution conditional on the generated rationale; they do not show that treatment-related learning is impossible or that the models ignored the OCT. The full 22-cell internal matrix is visualized in Figure 3.

### 3.3. Image-Responsive Biomarker Calls and Decision-Score Dissociation

**Figure 4:**
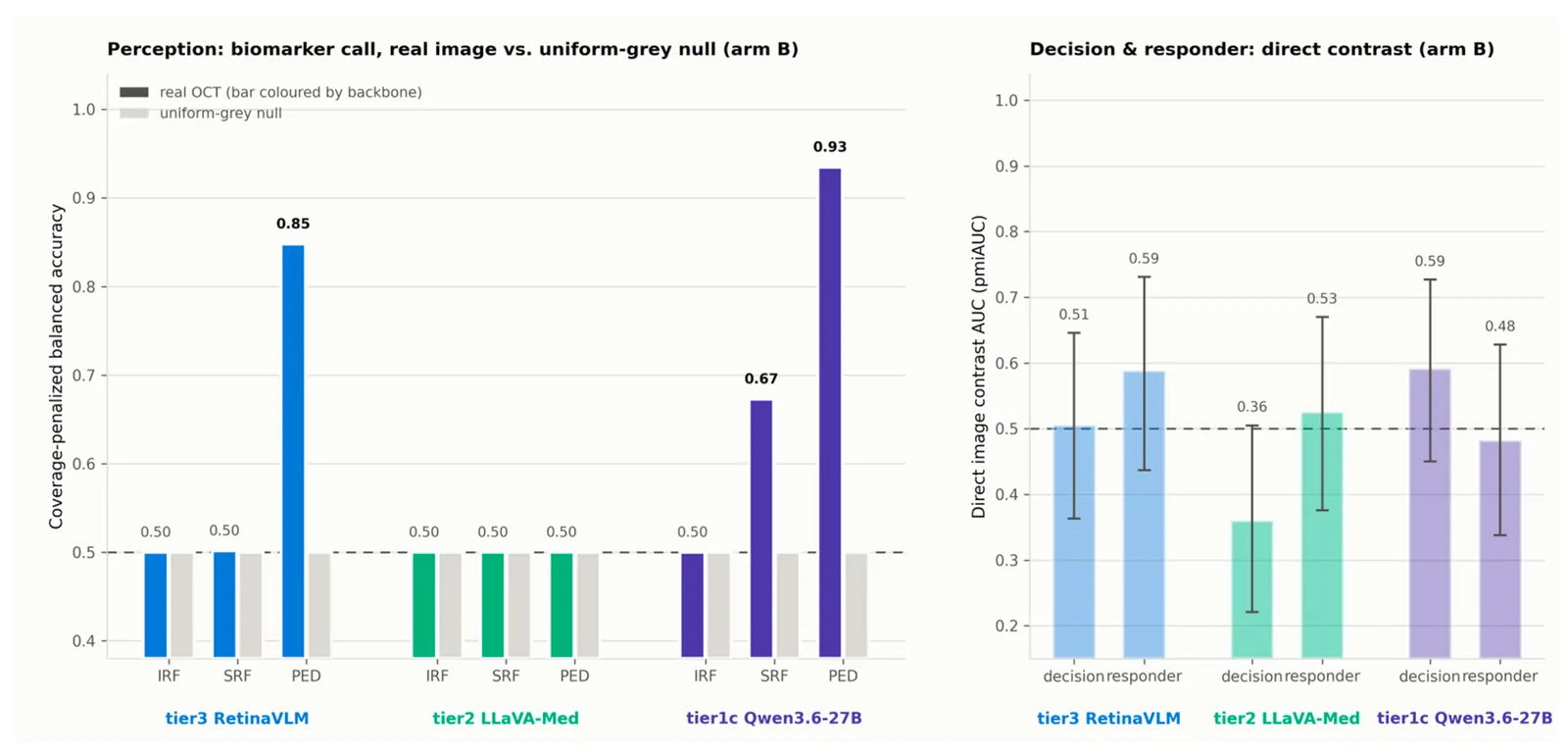
Biomarker-output and treatment-score dissociation. The left panel shows coverage-penalized balanced accuracy under arm B: PED is image-responsive for RetinaVLM (0.85) and Qwen (0.93), and SRF is image-responsive for Qwen (0.67), whereas LLaVA-Med remains at 0.50. Blue, green, and purple bars denote real-OCT outputs for RetinaVLM, LLaVA-Med, and Qwen3.6-27B, respectively; grey bars denote the uniform-grey null, and the black legend swatch denotes the real-OCT category generically. The horizontal dashed line marks balanced accuracy = 0.5. The right panel summarizes rationale-conditioned direct treatment and responder contrasts. These scores do not capture image effects mediated through generated rationales.

The biomarker and decision readouts behaved differently. Under arm B, PED coverage-penalized balanced accuracy reached 0.85 for RetinaVLM and 0.93 for Qwen. SRF rose only on Qwen (0.67); RetinaVLM stayed at 0.50 for SRF, and LLaVA-Med stayed at 0.50 for every label. PED is therefore the only label that responded on both strong visual backbones. Replacing the OCT with the uniform-grey image reduced the deterministic PED balanced accuracy to 0.50, indicating that the real image changed the biomarker output. This is evidence of image responsiveness, not proof of pixel-level grounding or causal localization. PED received image-level label supervision in the generated rationale and is a structural RPE-elevation biomarker, not a fluid compartment. The decision readout, by contrast, showed no consistently positive direct image contribution after the rationale was fixed. Because the mediated image→rationale→answer path was not measured, the data do not support the claim that the model “ignored the entire image.” Table 3 summarizes the per-label biomarker readouts, and Figure 4 illustrates their dissociation from the treatment-related contrasts.

### 3.4. Exploratory Attention and Counterfactual Checks

The attention-KL and prognosis analyses are exploratory. A reduction in training KL from 2.09 to 0.9 indicates that the optimization target changed, but without held-out pixel-level annotations, reliability testing, or causal intervention, it does not establish faithful attention. Similar decision scores in arms B and C show only that this specific regularizer did not improve the reported direct-effect readout. Likewise, four-category ΔCST performance of approximately 0.26–0.36 does not establish an information-theoretic ceiling; it may reflect limited sample size, image selection, encoder capacity, preprocessing, or target noise. Arm D is further limited by unvalidated masks, exclusion of PED, subtle occlusions, and a synthetic “dry→stop” label that is not a universal clinical rule.

### 3.5. Internal Tabular Reference Comparison

A logistic-regression reference model using the five pre-treatment fields achieved an internal decision AUC of 0.731 (95% CI 0.603–0.846) and a responder AUC of 0.687 (95% CI 0.534–0.820), with the baseline CST contributing substantially. This establishes that those structured variables contain predictive information in the internal split, but it is not a “ceiling.” The VLM metadata-prompt conditions did not match the tabular reference under the reported analysis. The contrast is consistent with, but does not localize, a fusion or sample-efficiency limitation; alternative explanations include prompt representation, adapter capacity, optimization, calibration, and training-set size. Prior multimodal retinal studies show that image-plus-structured-data fusion can improve performance in some settings but may lose performance under external shift [19,20].

### 3.6. Cross-Site Distribution-Shift Stress Test

**Figure 5:**
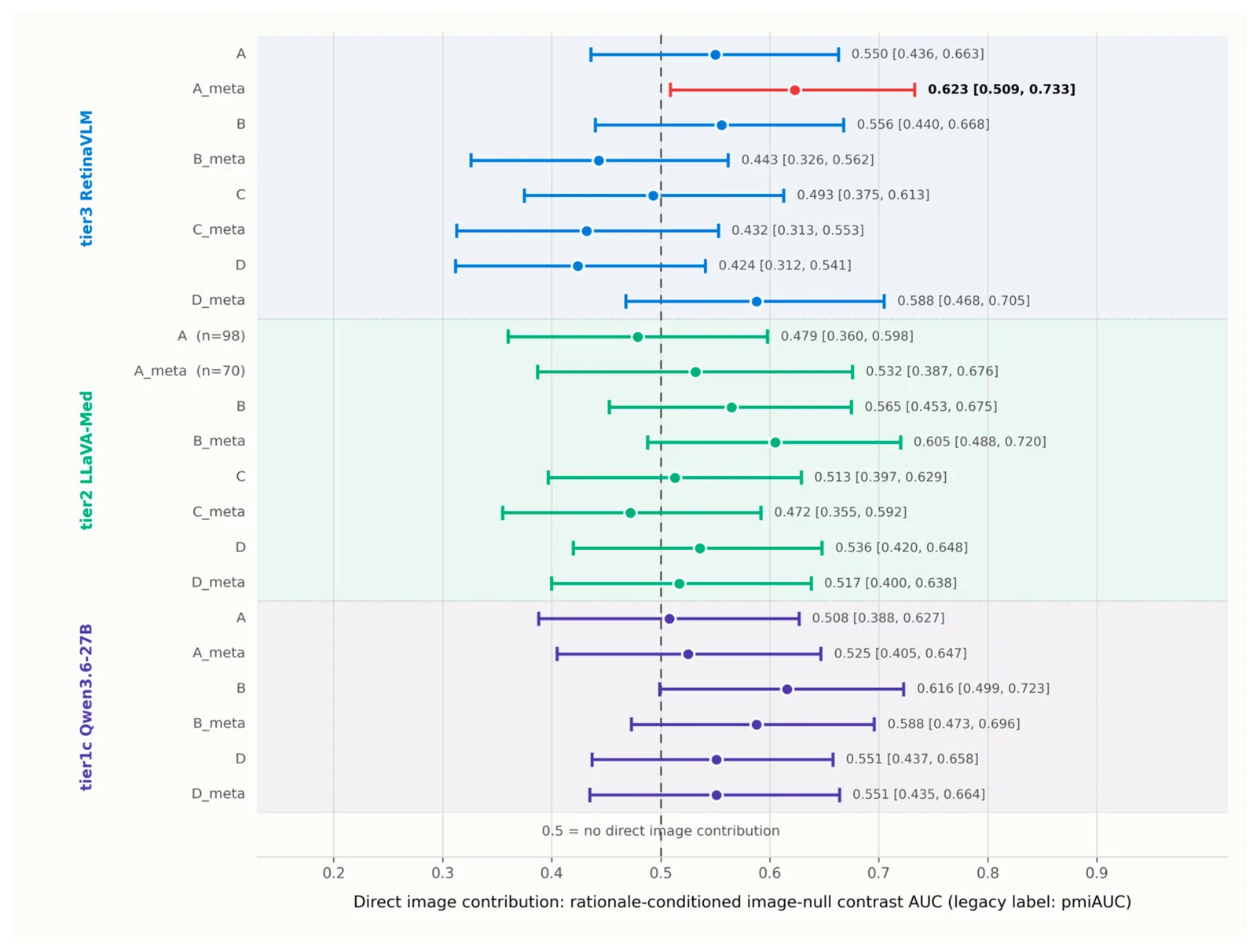
Cross-site rationale-conditioned direct-contrast AUCs for all 22 cells with bootstrap 95% confidence intervals. The embedded graphic retains the legacy label pmiAUC. The vertical dashed line marks AUC = 0.5. The red point and error bar and the bold label identify the single nominal cell whose 95% CI lies wholly above 0.5. Twenty-one intervals include 0.5; RetinaVLM A_meta is the single nominal exception (0.623 [0.509, 0.733]) and is not significant after Benjamini–Hochberg adjustment (q = 0.57). The pooled analysis is an exploratory distribution-shift stress test.

The APTOS-trained adapters were applied unchanged to the 100-eye second-site cohort. Under the rationale-conditioned uniform-grey contrast, 21 of 22 decision intervals included AUC 0.5. One interval nominally excluded 0.5 (RetinaVLM A_meta, 0.623 [0.509, 0.733]), but it was not significant after Benjamini–Hochberg adjustment across the 22-cell family (q = 0.57) and arose in an untrained zero-shot cell. No cell showed an FDR-significant direct image contribution in the aligned direction. These results are interpreted as an exploratory stress test under substantial clinical and technical heterogeneity, not as proof of chance performance or formal clinical replication. Complete cell-wise decision results are presented in Table 4 and Figure 5.

**Figure 6:**
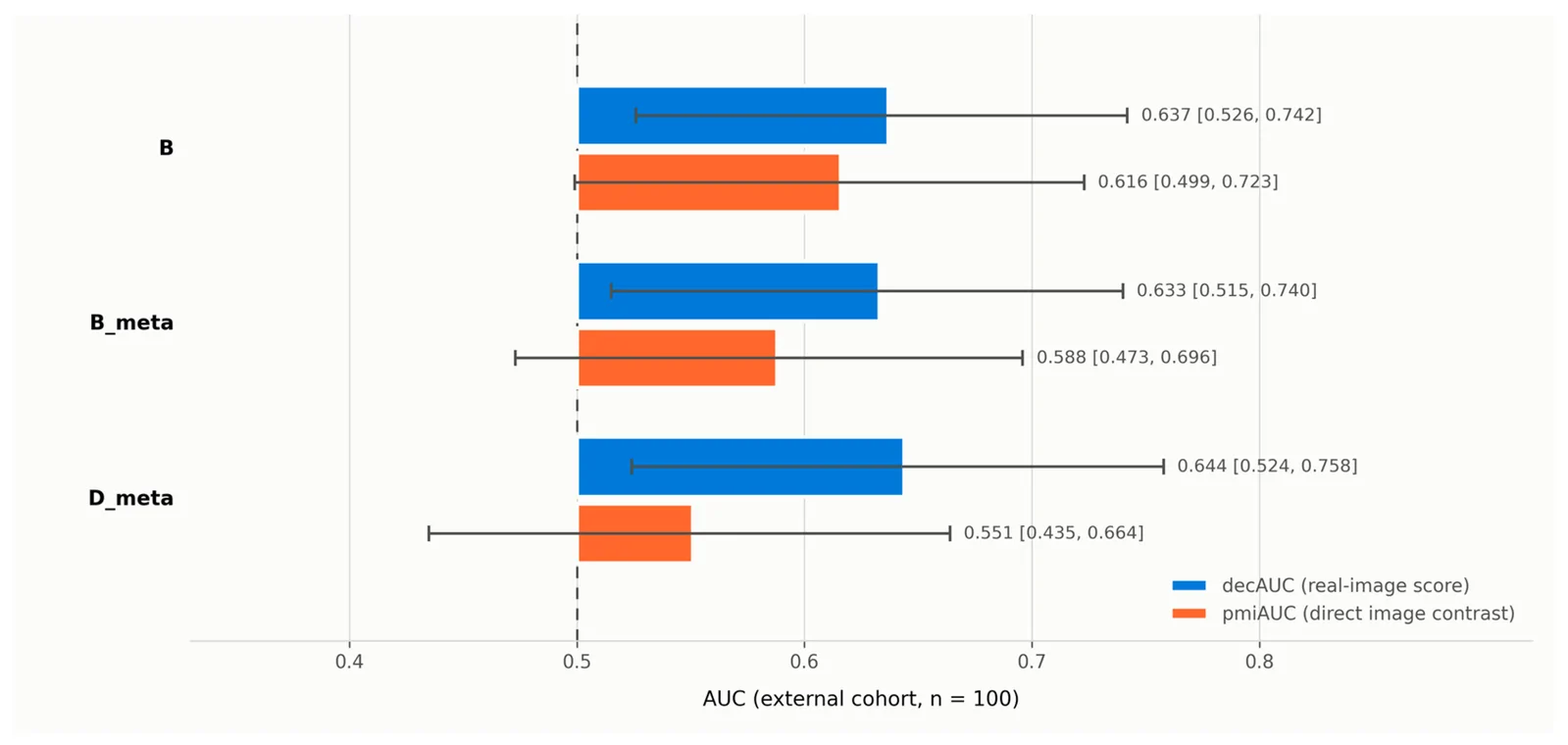
Raw real-image AUC versus rationale-conditioned direct-contrast AUC in selected Qwen cells. Blue bars denote real-image AUCs and orange bars denote direct-contrast AUCs; horizontal error bars are 95% confidence intervals, and the vertical dashed line marks AUC = 0.5. Differences are compatible with contributions from prompts, generated rationale, image processing, or their interaction. They do not validate the audit as a uniquely correct metric, prove the absence of mediated image effects, or identify a specific shortcut mechanism. All displayed intervals include AUC 0.5.

Selected Qwen cells showed higher raw real-image AUCs than their rationale-conditioned direct-contrast AUCs. This divergence is compatible with contributions from prompt or generated-text context, but it does not prove that raw performance was a text-prior artifact or that the image had no mediated effect. The Qwen arm B direct-contrast estimate of 0.616 (95% CI 0.499–0.723) is imprecise and is described as inconclusive. Raw-versus-contrast differences are reported descriptively; no causal source is assigned because prompt, model-scale, data-size, device, disease, and regimen factors were not independently manipulated. Selected raw real-image versus direct-contrast comparisons are shown in Figure 6.

**Figure 7:**
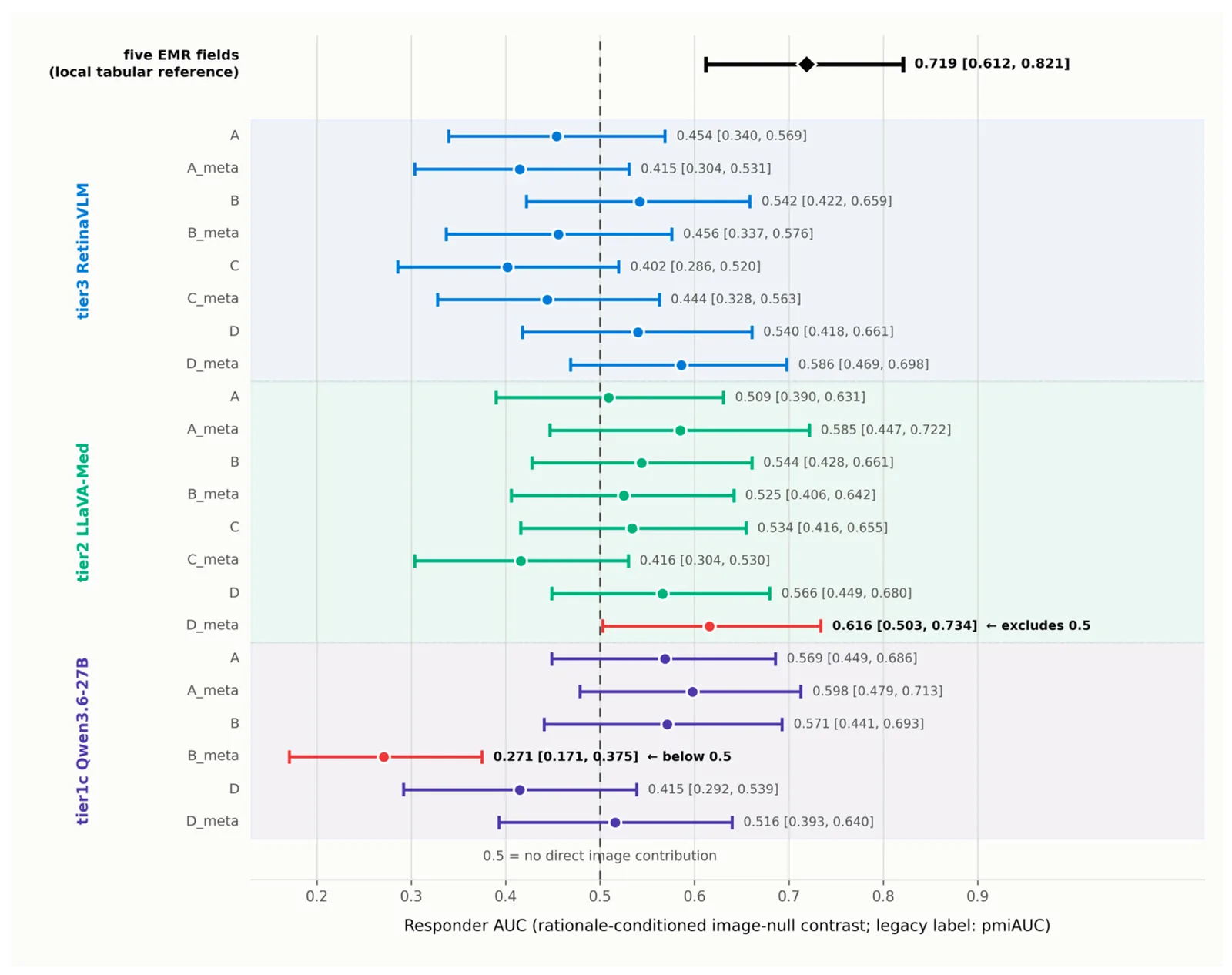
Local tabular responder reference and VLM responder contrasts in the second-site cohort. Colored circles denote VLM point estimates and horizontal lines denote 95% confidence intervals; blue, green, and purple identify RetinaVLM, LLaVA-Med, and Qwen3.6-27B, respectively. The black diamond and horizontal line denote the local tabular reference, and the vertical dashed line marks AUC = 0.5. Red points/error bars and bold labels identify the two nominal intervals excluding 0.5; neither remained significant after adjustment. The local tabular responder AUC is 0.719 (95% CI 0.612–0.821). Among 22 VLM responder cells, 20 intervals include 0.5; LLaVA-Med D_meta is nominally above 0.5 but not significant after adjustment (q = 0.47), and Qwen B_meta is inversely aligned. The comparison does not identify a specific fusion mechanism.

**Figure 8:**
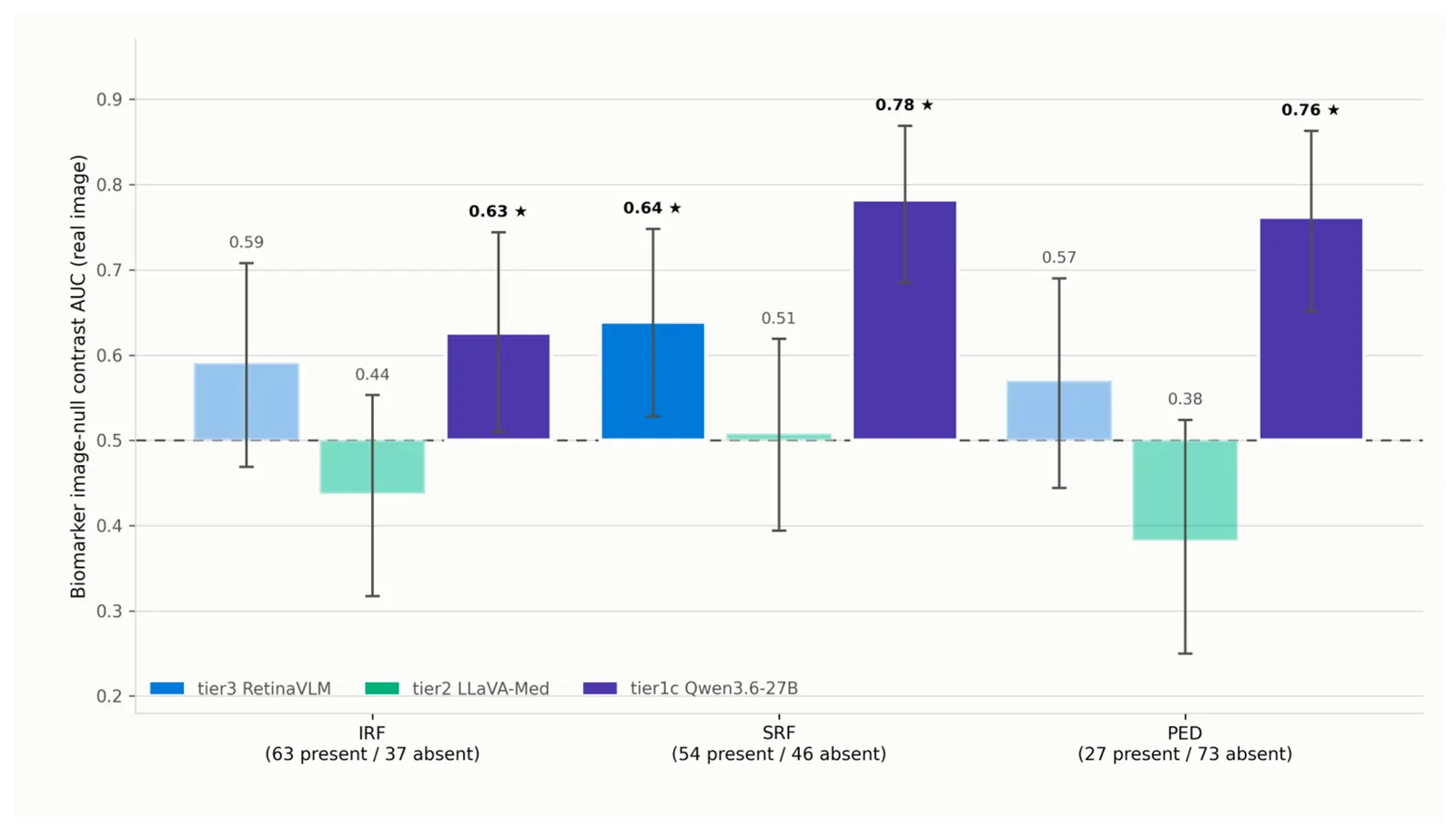
External per-label biomarker image-contrast AUCs by backbone. Light blue, mint green, and purple bars denote RetinaVLM, LLaVA-Med, and Qwen3.6-27B, respectively. The horizontal dashed line marks AUC = 0.5, and ★ denotes a backbone–label cell whose 95% CI lies entirely above 0.5. Selected Qwen labels and RetinaVLM SRF show nominally positive image-responsive discrimination, while other backbone–label combinations are inconclusive. These values do not prove pixel-level localization or establish the information content of a single pre-treatment B-scan. External base rates (present/absent) are IRF 63/37, SRF 54/46, and PED 27/73.

The locally refitted five-field tabular model achieved a decision AUC of 0.611 (95% CI 0.501–0.718) and a responder AUC of 0.719 (95% CI 0.612–0.821). Because the model was developed and cross-validated within the second-site cohort, these values are local reference estimates rather than an external validation of the internal tabular model. The decision estimate is borderline and should not be described as robust. The responder comparison suggests a gap between tabular and VLM readouts under this cohort, but it does not establish an architectural mechanism. External biomarker AUCs were higher for selected Qwen labels, whereas RetinaVLM and LLaVA-Med were closer to 0.5. These label-specific results support image responsiveness in selected cells but not a general claim that future-treatment information is absent from a single image. The local tabular comparison and responder results are summarized in Table 5 and Figure 7; external per-label biomarker results are shown in Figure 8.

## 4. Discussion

### 4.1. Scope and Interpretation of the Audit Protocol

The contribution of this study is an ophthalmology-specific audit configuration that makes one estimand explicit: the controlled direct contribution of the real image to a terminal score after the generated rationale is fixed. The method is not a new causal estimator, statistical framework, or debiasing algorithm. Option-order symmetry, bootstrap intervals, a text-sensitivity GATE, biomarker readouts, and tabular references are useful engineering checks, but they do not make the protocol self-validating. The generated rationale is an observed model output whose fidelity to latent computation was not established; gradient-based attribution, attention visualization, concept probing, counterfactual explanation, and the present image-replacement contrast should therefore be viewed as complementary tools [24,25,26,27,28,29,30,31,32,33,34,35,36,37,38]. None alone establishes causal grounding or clinical validity.

### 4.2. What the Results Do and Do Not Establish

The data support a narrower conclusion than an information ceiling. Under the tested checkpoints, prompts, adapters, and uniform-grey null, no consistently positive direct image contribution was demonstrated after conditioning on the generated rationale. The result may reflect limited extractable signal, encoder or preprocessing limitations, small-sample adaptation, target heterogeneity, global response to an out-of-distribution null, or transfer of image information into the rationale before the image was replaced. The confidence intervals do not exclude moderate effects in several cells, and no prespecified equivalence margin was available. The tabular reference indicates that structured pre-treatment variables contain predictive information in the internal split, but failure of prompt-based fusion does not localize an architectural defect.

### 4.3. Engineering and Clinical Design Implications

Several clinical and engineering implications follow. End-task accuracy should be accompanied by modality ablation, output coverage, calibration, and an explicit definition of the estimand. The present audit does not establish either clinical utility or clinical nonutility because the primary label was a retrospective challenge target, not an independently adjudicated treatment decision, and the input omitted serial OCT, visual-acuity trajectories, prior injections, treatment interval, prior response, comorbidities, and patient preference. Recent Korean real-world data in previously treated nAMD eyes showed that short-term anatomical response after faricimab switching was associated with baseline CST, baseline SRF status, and prior brolucizumab exposure, reinforcing the need for disease- and regimen-specific models that represent treatment history and baseline phenotype [50]. Prospective retina-specialist adjudication, reader comparison, calibration, and decision-curve analysis are required before either the audit or the evaluated models can be interpreted as clinical decision support.

The cross-site analysis also cannot identify a single cause of distribution shift. Potential contributors include OCT device and acquisition differences, disease-subtype composition, drug and regimen heterogeneity, prior treatment exposure, endpoint prevalence, and the selection of one representative B-scan. Because these factors were not independently manipulated, the study does not prescribe a specific robustness intervention. Systematic ablations of prompt wording, model scale, fine-tuning-data size, and null construction, together with conventional CNN/ViT, full-volume OCT, image–tabular fusion, and masked retina-specialist comparators, are planned for an expanded follow-up study.

### 4.4. Data Privacy and Secure Imaging Pipelines

Although both cohorts were de-identified, medical images remain potentially sensitive throughout storage, transfer, model development, and multicenter collaboration. Clinical AI pipelines should combine de-identification with encryption at rest and in transit, role-based access control, audit logging, and institutionally governed data access. Privacy-preserving image-encryption methods may provide an additional safeguard. For example, Song et al. proposed batch-image encryption using cross-image permutation and bidirectional diffusion for heterogeneous image sizes [51]. Such methods were not evaluated in this study; their computational burden, interoperability, reversibility, and preservation of diagnostically relevant OCT features require ophthalmic validation before deployment.

### 4.5. Limitations and Future Validation

This study has substantial limitations. The internal test set contains 69 eyes, the second-site stress-test cohort contains 100 eyes from one site, and one split and one training realization were analyzed; bootstrap intervals do not include retraining variability, and no prespecified equivalence margin or formal sample-size design was available. The primary endpoint is a dataset-provided retrospective label without blinded retina-specialist re-adjudication, and a single pre-treatment B-scan does not reproduce routine anti-VEGF management. The second-site cohort is heterogeneous in disease and therapy and includes two dexamethasone/Ozurdex cases. The primary contrast fixes the real-image rationale, omits rationale-mediated image effects, and assumes neither that the generated rationale is faithful nor that it captures latent computation. The uniform-grey input is an out-of-distribution single null; no in-domain wrong-patient OCT, null ensemble, or same-backbone image-determined positive control was tested. Conventional CNN/ViT, quantitative OCT, full-volume, fused multimodal, oracle-biomarker, and human-reader comparators were not included. Nominal confidence intervals and within-family FDR adjustment do not establish equivalence. LLaVA-Med had substantial missing generations, making complete-case estimates vulnerable to selection bias. Fluid masks lack APTOS pixel-level ground truth, omit PED, and were not independently graded; the synthetic fluid-removal target is not a universal clinical counterfactual. Exact historical software build numbers and hardware identifiers were not preserved. These limitations motivate a prospective, repeated-seed, patient-level study with a 2 × 2 image × rationale design, multiple in-domain nulls, clinically adjudicated endpoints, established explainability comparators, and disease-specific longitudinal inputs.

## 5. Conclusions

Across the tested VLM checkpoints and parameter-efficient adaptation settings, we did not identify a consistently positive rationale-conditioned direct contribution of the OCT image to the final continue/stop score under a uniform-grey single-null intervention. Selected backbones produced image-responsive biomarker outputs, but this does not establish pixel-level grounding or faithful use of those biomarkers in the treatment score. Because the analysis fixes a potential mediator, does not validate rationale fidelity, uses one out-of-distribution null, and lacks conventional image-model and human comparators, it cannot show that the models ignored OCT, establish absence of total image information, or demonstrate an information-theoretic single-image ceiling. The second-site cohort is an exploratory, heterogeneous distribution-shift stress test. The principal contribution is therefore methodological and cautionary: clinical VLM evaluation should distinguish raw end-task performance, rationale-conditioned direct image effects, rationale-mediated effects, output coverage, calibration, and transportability. Factorial imaging × rationale experiments, in-domain null controls, established explanation methods, conventional OCT comparators, and prospectively adjudicated longitudinal studies are priorities for future work.

## Figures and Tables

**Table 1:** Internal zero-shot rationale-conditioned direct-image contrasts (*n* = 69; LLaVA-Med A_meta complete-case *n* = 52). The embedded label pmiAUC denotes the AUC of the uniform-grey single-null image contrast. An interval including 0.5 is inconclusive rather than equivalent to no discrimination; Qwen arm A is inversely aligned (0.354 [0.224, 0.493]).

Backbone	Arm	|Δ|	Real AUC [95% CI]	Direct-Contrast AUC [95% CI]
tier3 (RetinaVLM)	A	0.275	0.334 [0.202, 0.477]	0.368 [0.230, 0.518]
tier3 (RetinaVLM)	A_meta	0.199	0.415 [0.267, 0.573]	0.423 [0.281, 0.574]
tier2 (LLaVA-Med)	A	0.007	0.445 [0.298, 0.590]	0.452 [0.316, 0.591]
tier2 (LLaVA-Med)	A_meta ^†^	0.003	0.437 [0.264, 0.605]	0.490 [0.322, 0.659]
tier1c (Qwen3.6-27B)	A	0.474	0.471 [0.333, 0.615]	0.354 [0.224, 0.493]
tier1c (Qwen3.6-27B)	A_meta	0.440	0.512 [0.358, 0.668]	0.367 [0.237, 0.502]

^†^ LLaVA-Med produced empty newline-only outputs for 17/69 eyes. These cases were excluded from the complete-case AUC, yielding *n* = 52.

**Table 2:** Internal results for all 16 trained cells (*n* = 69). Together with the six zero-shot cells in Table 1, these constitute the 22-cell design. Real AUC is the AUC of the terminal score with the real image; Direct-contrast AUC is the rationale-conditioned real-versus-grey image contrast (legacy label pmiAUC); |Δ| is the mean absolute score change; Resp AUC is the corresponding responder contrast. Continue rates near 1.00 indicate hard-output collapse. Bootstrap 95% CIs used 5000 patient-clustered replicates (seed 0). An interval including 0.5 is inconclusive.

Backbone	Arm	Cont.	BM	Real AUC [95% CI]	Direct-Contrast AUC [95% CI]	|Δ|	Resp AUC [95% CI]
tier3 (RetinaVLM)	B	1.00	0.778	0.521 [0.379, 0.659]	0.505 [0.363, 0.646]	0.302	0.588 [0.437, 0.731]
tier3 (RetinaVLM)	B_meta	1.00	0.797	0.407 [0.275, 0.547]	0.411 [0.278, 0.552]	0.048	0.593 [0.442, 0.735]
tier3 (RetinaVLM)	C	1.00	0.768	0.520 [0.374, 0.666]	0.492 [0.347, 0.634]	0.104	0.375 [0.211, 0.543]
tier3 (RetinaVLM)	C_meta	1.00	0.797	0.489 [0.347, 0.637]	0.493 [0.348, 0.641]	0.083	0.434 [0.297, 0.579]
tier3 (RetinaVLM)	D	0.78	0.656	0.475 [0.329, 0.621]	0.531 [0.390, 0.671]	0.118	0.393 [0.232, 0.559]
tier3 (RetinaVLM)	D_meta	0.94	0.764	0.359 [0.223, 0.496]	0.425 [0.288, 0.566]	0.087	0.425 [0.271, 0.587]
tier2 (LLaVA-Med)	B	1.00	0.691	0.343 [0.205, 0.488]	0.360 [0.221, 0.505]	0.004	0.525 [0.376, 0.670]
tier2 (LLaVA-Med)	B_meta	1.00	0.744	0.422 [0.285, 0.565]	0.480 [0.326, 0.637]	0.006	0.541 [0.389, 0.688]
tier2 (LLaVA-Med)	C	1.00	0.691	0.407 [0.276, 0.540]	0.398 [0.265, 0.534]	0.000	0.458 [0.309, 0.606]
tier2 (LLaVA-Med)	C_meta	1.00	0.691	0.450 [0.303, 0.596]	0.494 [0.348, 0.636]	0.014	0.388 [0.240, 0.534]
tier2 (LLaVA-Med)	D	1.00	0.744	0.397 [0.261, 0.540]	0.380 [0.238, 0.532]	0.004	0.588 [0.416, 0.752]
tier2 (LLaVA-Med)	D_meta	1.00	0.744	0.456 [0.311, 0.606]	0.476 [0.329, 0.620]	0.004	0.625 [0.479, 0.767]
tier1c (Qwen3.6-27B)	B	1.00	0.850	0.590 [0.448, 0.725]	0.591 [0.450, 0.727]	0.158	0.482 [0.338, 0.628]
tier1c (Qwen3.6-27B)	B_meta	1.00	0.850	0.321 [0.194, 0.461]	0.371 [0.233, 0.517]	0.668	0.375 [0.223, 0.538]
tier1c (Qwen3.6-27B)	D	0.88	0.797	0.476 [0.337, 0.624]	0.482 [0.341, 0.626]	0.204	0.510 [0.362, 0.660]
tier1c (Qwen3.6-27B)	D_meta	0.99	0.850	0.424 [0.285, 0.566]	0.443 [0.301, 0.583]	0.519	0.562 [0.395, 0.722]

**Table 3:** Per-label biomarker readouts. PED is a structural RPE-elevation biomarker rather than a fluid compartment. Internal values are coverage-penalized balanced accuracy under arm B; external values are per-label image-contrast AUCs under arm B with bootstrap 95% CIs. Grey-image collapse demonstrates image responsiveness but not lesion localization or faithful causal use. External base rates (present/absent) are IRF 63/37, SRF 54/46, and PED 27/73.

	Internal BalAcc (Arm B)			External Image-Null AUC		
Backbone	PED	PED (blank)	SRF	IRF	SRF	PED
tier3 (RetinaVLM)	0.85	0.50	0.50	0.59 [0.47, 0.71]	0.64 [0.53, 0.75] ★	0.57 [0.44, 0.69]
tier2 (LLaVA-Med)	0.50	0.50	0.50	0.44 [0.32, 0.55]	0.51 [0.39, 0.62]	0.38 [0.25, 0.52]
tier1c (Qwen3.6-27B)	0.93	0.50	0.67	0.63 [0.51, 0.74] ★	0.78 [0.69, 0.87] ★	0.76 [0.65, 0.86] ★

★ indicates a backbone–label cell whose 95% CI lies entirely above AUC 0.5. Cells without ★ are inconclusive. The symbol denotes nominal discrimination on the biomarker task and does not establish pixel-level grounding.

**Table 4:** Cross-site decision readouts for all 22 cells. Direct-contrast AUC denotes the rationale-conditioned uniform-grey image contrast (legacy label pmiAUC). Bootstrap 95% CIs used 5000 patient-clustered replicates (seed 0), and the output coverage is shown for each cell. LLaVA-Med produced newline-degenerate outputs in 30/100 A_meta cases (complete-case *n* = 70) and 2/100 A cases (*n* = 98); all other cells had 100/100 coverage. An interval including 0.5 is inconclusive and is not described as chance performance.

Backbone	Arm (Coverage)	DecAUC [95% CI]	Direct-Contrast AUC [95% CI]
tier3 (RetinaVLM)	A (100/100)	0.567 [0.452, 0.678]	0.550 [0.436, 0.663]
tier3 (RetinaVLM)	A_meta (100/100)	0.570 [0.454, 0.686]	0.623 [0.509, 0.733] ^‡^
tier3 (RetinaVLM)	B (100/100)	0.552 [0.435, 0.668]	0.556 [0.440, 0.668]
tier3 (RetinaVLM)	B_meta (100/100)	0.442 [0.322, 0.563]	0.443 [0.326, 0.562]
tier3 (RetinaVLM)	C (100/100)	0.475 [0.358, 0.598]	0.493 [0.375, 0.613]
tier3 (RetinaVLM)	C_meta (100/100)	0.437 [0.321, 0.560]	0.432 [0.313, 0.553]
tier3 (RetinaVLM)	D (100/100)	0.436 [0.325, 0.551]	0.424 [0.312, 0.541]
tier3 (RetinaVLM)	D_meta (100/100)	0.604 [0.483, 0.721]	0.588 [0.468, 0.705]
tier2 (LLaVA-Med)	A (98/100)	0.527 [0.408, 0.650]	0.479 [0.360, 0.598]
tier2 (LLaVA-Med)	A_meta (70/100)	0.599 [0.456, 0.738]	0.532 [0.387, 0.676]
tier2 (LLaVA-Med)	B (100/100)	0.538 [0.421, 0.653]	0.565 [0.453, 0.675]
tier2 (LLaVA-Med)	B_meta (100/100)	0.388 [0.276, 0.499]	0.605 [0.488, 0.720]
tier2 (LLaVA-Med)	C (100/100)	0.534 [0.415, 0.646]	0.513 [0.397, 0.629]
tier2 (LLaVA-Med)	C_meta (100/100)	0.433 [0.315, 0.550]	0.472 [0.355, 0.592]
tier2 (LLaVA-Med)	D (100/100)	0.543 [0.427, 0.657]	0.536 [0.420, 0.648]
tier2 (LLaVA-Med)	D_meta (100/100)	0.502 [0.385, 0.617]	0.517 [0.400, 0.638]
tier1c (Qwen3.6-27B)	A (100/100)	0.553 [0.434, 0.671]	0.508 [0.388, 0.627]
tier1c (Qwen3.6-27B)	A_meta (100/100)	0.474 [0.351, 0.599]	0.525 [0.405, 0.647]
tier1c (Qwen3.6-27B)	B (100/100)	0.637 [0.526, 0.742]	0.616 [0.499, 0.723]
tier1c (Qwen3.6-27B)	B_meta (100/100)	0.633 [0.515, 0.740]	0.588 [0.473, 0.696]
tier1c (Qwen3.6-27B)	D (100/100)	0.585 [0.466, 0.699]	0.551 [0.437, 0.658]
tier1c (Qwen3.6-27B)	D_meta (100/100)	0.644 [0.524, 0.758]	0.551 [0.435, 0.664]

^‡^ Nominal cell-wise intervals are unadjusted for multiplicity. Exactly one of the 22 intervals excludes 0.5 (RetinaVLM A_meta); after Benjamini–Hochberg adjustment it is not significant (q = 0.57), and no cell survives adjustment in the aligned direction. Such intervals are exploratory and do not establish transportable clinical validity.

**Table 5:** Local second-site tabular reference compared with selected VLM metadata-prompt readouts. The tabular model was refitted and cross-validated within the second-site cohort; it is therefore a local reference rather than an external validation estimate or performance ceiling. Among 22 responder contrasts, 20 intervals include 0.5. LLaVA-Med D_meta is nominally above 0.5 (0.616 [0.503, 0.734]) but is not significant after adjustment (q = 0.47); Qwen B_meta is inversely aligned (0.271 [0.171, 0.375]).

Target	Local Tabular AUC [95% CI]	Selected VLM Readout [95% CI]
decision	0.611 [0.501, 0.718]	tier3 0.570 [0.454, 0.686]; tier2 0.599 [0.456, 0.738]; tier1c 0.474 [0.351, 0.599]
responder	0.719 [0.612, 0.821]	tier3 0.445 [0.332, 0.565]; tier2 0.409 [0.276, 0.548]; tier1c 0.520 [0.396, 0.641]. Rationale-conditioned direct-contrast AUC, all 22 cells: 20/22 intervals include 0.5; nominal exceptions were tier2 D_meta 0.616 [0.503, 0.734] and tier1c B_meta 0.271 [0.171, 0.375].

## Data Availability

The internal cohort is the de-identified APTOS-2021 anti-VEGF challenge dataset, obtainable through the official challenge platform under its data-use terms. The second-site stress-test cohort is subject to institutional data-sharing restrictions; de-identified data may be available from the corresponding author on reasonable request and subject to ethics approval and institutional agreements. The Supplementary Materials section provides public repository mappings, checkpoint-provenance limitations, preprocessing, prompts, adapter settings, and the scoring procedure. De-identified aggregate outputs and analysis scripts may be requested from the corresponding author where permitted. Raw second-site images are not publicly deposited because the consent waiver and institutional governance do not authorize unrestricted public release.

## Supplementary Materials

The following supporting information can be downloaded at: https://www.mdpi.com/article/10.3390/bioengineering13080912/s1. The following supporting information accompanies this manuscript: Supplementary Methods S1–S9; Table S1, public checkpoint mappings and provenance status; Table S2, adaptation and training configuration; Table S3, prompt and scoring schema; and Table S4, reproducibility and security scope.

## References

[1] Guymer R.H., Markey C.M., McAllister I.L., Gillies M.C., Hunyor A.P., Arnold J.J. (2019). Tolerating Subretinal Fluid in Neovascular Age-Related Macular Degeneration Treated with Ranibizumab Using a Treat-and-Extend Regimen: FLUID Study 24-Month Results. Ophthalmology.

[2] Riedl S., Vogl W.D., Waldstein S.M., Schmidt-Erfurth U., Bogunović H. (2022). Impact of Intra- and Subretinal Fluid on Vision Based on Volume Quantification in the HARBOR Trial. Ophthalmol. Retin..

[3] Bogunović H., Mares V., Reiter G.S., Schmidt-Erfurth U. (2022). Predicting treat-and-extend outcomes and treatment intervals in neovascular age-related macular degeneration from retinal optical coherence tomography using artificial intelligence. Front. Med..

[4] Schmidt-Erfurth U., Bogunović H., Sadeghipour A., Schlegl T., Langs G., Gerendas B.S., Osborne A., Waldstein S.M. (2018). Machine Learning to Analyze the Prognostic Value of Current Imaging Biomarkers in Neovascular Age-Related Macular Degeneration. Ophthalmol. Retin..

[5] Vogl W.D., Waldstein S.M., Gerendas B.S., Schlegl T., Langs G., Schmidt-Erfurth U. (2017). Analyzing and Predicting Visual Acuity Outcomes of Anti-VEGF Therapy by a Longitudinal Mixed Effects Model of Imaging and Clinical Data. Investig. Ophthalmol. Vis. Sci..

[6] Banerjee I., de Sisternes L., Hallak J.A., Leng T., Osborne A., Rosenfeld P.J., Gregori G., Durbin M., Rubin D. (2020). Prediction of age-related macular degeneration disease using a sequential deep learning approach on longitudinal SD-OCT imaging biomarkers. Sci. Rep..

[7] Wong W.L., Su X., Li X., Cheung C.M.G., Klein R., Cheng C.Y., Wong T.Y. (2014). Global prevalence of age-related macular degeneration and disease burden projection for 2020 and 2040: A systematic review and meta-analysis. Lancet Glob. Health.

[8] Bogunović H., Venhuizen F., Klimscha S., Apostolopoulos S., Bab-Hadiashar A., Bagci U., Beg M.F., Bekalo L., Chen Q., Ciller C. (2019). RETOUCH: The Retinal OCT Fluid Detection and Segmentation Benchmark and Challenge. IEEE Trans. Med. Imaging.

[9] Roy A.G., Conjeti S., Karri S.P.K., Sheet D., Katouzian A., Wachinger C., Navab N. (2017). ReLayNet: Retinal Layer and Fluid Segmentation of Macular Optical Coherence Tomography using Fully Convolutional Network. Biomed. Opt. Express.

[10] Yeh T.C., Luo A.C., Deng Y.S., Lee Y.H., Chen S.J., Chang P.H., Lin C.J., Tai M.C., Chou Y.B. (2022). Prediction of treatment outcome in neovascular age-related macular degeneration using a novel convolutional neural network. Sci. Rep..

[11] Han J.M., Han J., Ko J., Jung J., Park J.I., Hwang J.S., Yoon J., Jung J.H., Hwang D.D. (2024). Anti-VEGF treatment outcome prediction based on optical coherence tomography images in neovascular age-related macular degeneration using a deep neural network. Sci. Rep..

[12] Zhang W., Chotcomwongse P., Li Y., Xu P., Yao R., Zhou L., Zhou Y., Feng H., Zhou Q., Wang X. (2026). Predicting Diabetic Macular Edema Treatment Responses Using OCT: Dataset and Methods of APTOS Competition. Med. Image Anal..

[13] Holland R., Taylor T.R.P., Holmes C., Riedl S., Mai J., Patsiamanidi M., Mitsopoulou D., Hager P., Müller P., Scholl H.P.N. (2025). Specialized curricula for training vision-language models in retinal image analysis. npj Digit. Med..

[14] Li C., Wong C., Zhang S., Usuyama N., Liu H., Yang J., Naumann T., Poon H., Gao J. (2023). LLaVA-Med: Training a Large Language-and-Vision Assistant for Biomedicine in One Day. Proceedings of the Advances in Neural Information Processing Systems (NeurIPS).

[15] Moor M., Huang Q., Wu S., Yasunaga M., Dalmia Y., Zakka C., Reis E.P., Rajpurkar P., Leskovec J. (2023). Med-Flamingo: A Multimodal Medical Few-Shot Learner. Proceedings of the 3rd Machine Learning for Health Symposium (ML4H).

[16] Zhang X., Wu C., Zhang Y., Xie W., Wang Y. (2023). Knowledge-enhanced visual-language pre-training on chest radiology images. Nat. Commun..

[17] Wang M.H. (2025). Explainable Artificial Intelligence Framework for Predicting Treatment Outcomes in Age-Related Macular Degeneration. Sensors.

[18] Qwen Team (2026). Qwen3.6-27B Model Card and Checkpoint. Hugging Face. https://huggingface.co/Qwen/Qwen3.6-27B.

[19] Bhak Y., Lee Y.H., Kim J., Lee K., Lee D., Jang E.C., Jang E., Lee C.S., Kang E.S., Park S. (2025). Diagnosis of Chronic Kidney Disease Using Retinal Imaging and Urine Dipstick Data: Multimodal Deep Learning Approach. JMIR Med. Inform..

[20] Ahn S., Shin J., Song S.J., Yoon W.T., Sagong M., Jeong A., Kim J.H., Yu H.G. (2023). Neurologic Dysfunction Assessment in Parkinson Disease Based on Fundus Photographs Using Deep Learning. JAMA Ophthalmol..

[21] Xia P., Chen Z., Tian J., Gong Y., Hou R., Xu Y., Wu Z., Fan Z., Zhou Y., Zhu K. (2024). CARES: A Comprehensive Benchmark of Trustworthiness in Medical Vision Language Models. Proceedings of the Advances in Neural Information Processing Systems (NeurIPS), Datasets and Benchmarks Track.

[22] Chang A., Huang L., Bhatia P., Kass-Hout T., Ma F., Xiao C. (2025). MedHEval: Benchmarking Hallucinations and Mitigation Strategies in Medical Large Vision-Language Models. arXiv.

[23] Buckley T., Diao J.A., Rajpurkar P., Rodman A., Manrai A.K. (2023). Multimodal Foundation Models Exploit Text to Make Medical Image Predictions. arXiv.

[24] Turpin M., Michael J., Perez E., Bowman S.R. (2023). Language Models Don’t Always Say What They Think: Unfaithful Explanations in Chain-of-Thought Prompting. Proceedings of the Advances in Neural Information Processing Systems (NeurIPS).

[25] Lanham T., Chen A., Radhakrishnan A., Steiner B., Denison C., Hernandez D., Li D., Durmus E., Hubinger E., Kernion J. (2023). Measuring Faithfulness in Chain-of-Thought Reasoning. arXiv.

[26] Chen Y., Benton J., Radhakrishnan A., Uesato J., Denison C., Schulman J., Somani A., Hase P., Wagner M., Roger F. (2025). Reasoning Models Don’t Always Say What They Think. arXiv.

[27] Balasubramanian S., Basu S., Feizi S. (2025). A Closer Look at Bias and Chain-of-Thought Faithfulness of Large (Vision) Language Models. arXiv.

[28] Atanasova P., Camburu O.M., Lioma C., Lukasiewicz T., Simonsen J.G., Augenstein I. (2023). Faithfulness Tests for Natural Language Explanations. Proceedings of the 61st Annual Meeting of the Association for Computational Linguistics (Volume 2: Short Papers).

[29] Jacovi A., Goldberg Y. (2020). Towards Faithfully Interpretable NLP Systems: How Should We Define and Evaluate Faithfulness?. Proceedings of the 58th Annual Meeting of the Association for Computational Linguistics (ACL).

[30] Holtzman A., West P., Shwartz V., Choi Y., Zettlemoyer L. (2021). Surface Form Competition: Why the Highest Probability Answer Is Not Always Right. Proceedings of the 2021 Conference on Empirical Methods in Natural Language Processing (EMNLP).

[31] Zhao T.Z., Wallace E., Feng S., Klein D., Singh S. (2021). Calibrate Before Use: Improving Few-Shot Performance of Language Models. Proceedings of the 38th International Conference on Machine Learning (ICML).

[32] Jain S., Wallace B.C. (2019). Attention Is Not Explanation. Proceedings of the 2019 Conference of the North American Chapter of the Association for Computational Linguistics: Human Language Technologies (NAACL-HLT).

[33] Wiegreffe S., Pinter Y. (2019). Attention is not not Explanation. Proceedings of the EMNLP-IJCNLP.

[34] Abnar S., Zuidema W. (2020). Quantifying Attention Flow in Transformers. Proceedings of the 58th Annual Meeting of the Association for Computational Linguistics (ACL).

[35] Chefer H., Gur S., Wolf L. (2021). Transformer Interpretability Beyond Attention Visualization. Proceedings of the IEEE/CVF Conference on Computer Vision and Pattern Recognition (CVPR).

[36] Adebayo J., Gilmer J., Muelly M., Goodfellow I., Hardt M., Kim B. (2018). Sanity Checks for Saliency Maps. Proceedings of the Advances in Neural Information Processing Systems (NeurIPS).

[37] Arun N., Gaw N., Singh P., Chang K., Aggarwal M., Chen B., Hoebel K., Gupta S., Patel J., Gidwani M. (2021). Assessing the Trustworthiness of Saliency Maps for Localizing Abnormalities in Medical Imaging. Radiol. Artif. Intell..

[38] Saporta A., Gui X., Agrawal A., Pareek A., Truong S.Q.H., Nguyen C.D.T., Ngo V.D., Seekins J., Blankenberg F.G., Ng A.Y. (2022). Benchmarking saliency methods for chest X-ray interpretation. Nat. Mach. Intell..

[39] Geirhos R., Jacobsen J.H., Michaelis C., Zemel R., Brendel W., Bethge M., Wichmann F.A. (2020). Shortcut Learning in Deep Neural Networks. Nat. Mach. Intell..

[40] DeGrave A.J., Janizek J.D., Lee S.I. (2021). AI for Radiographic COVID-19 Detection Selects Shortcuts over Signal. Nat. Mach. Intell..

[41] Rudin C. (2019). Stop explaining black box machine learning models for high stakes decisions and use interpretable models instead. Nat. Mach. Intell..

[42] Ouyang L., Wu J., Jiang X., Almeida D., Wainwright C.L., Mishkin P., Zhang C., Agarwal S., Slama K., Ray A. (2022). Training Language Models to Follow Instructions with Human Feedback. Proceedings of the Advances in Neural Information Processing Systems (NeurIPS).

[43] Biderman D., Portes J., Ortiz J.J.G., Paul M., Greengard P., Jennings C., King D., Havens S., Chiley V., Frankle J. (2024). LoRA Learns Less and Forgets Less. arXiv.

[44] McCloskey M., Cohen N.J. (1989). Catastrophic Interference in Connectionist Networks: The Sequential Learning Problem. Psychology of Learning and Motivation.

[45] Kirkpatrick J., Pascanu R., Rabinowitz N., Veness J., Desjardins G., Rusu A.A., Milan K., Quan J., Ramalho T., Grabska-Barwinska A. (2017). Overcoming Catastrophic Forgetting in Neural Networks. Proc. Natl. Acad. Sci. USA.

[46] Luo Y., Yang Z., Meng F., Li Y., Zhou J., Zhang Y. (2023). An Empirical Study of Catastrophic Forgetting in Large Language Models During Continual Fine-tuning. arXiv.

[47] Chu T., Zhai Y., Yang J., Tong S., Xie S., Schuurmans D., Le Q.V., Levine S., Ma Y. (2025). SFT Memorizes, RL Generalizes: A Comparative Study of Foundation Model Post-training. Proceedings of the International Conference on Machine Learning (ICML).

[48] Li K., Wu Z., Peng K.C., Ernst J., Fu Y. (2018). Tell Me Where to Look: Guided Attention Inference Network. Proceedings of the IEEE Conference on Computer Vision and Pattern Recognition (CVPR).

[49] Ross A.S., Hughes M.C., Doshi-Velez F. (2017). Right for the Right Reasons: Training Differentiable Models by Constraining Their Explanations. Proceedings of the Twenty-Sixth International Joint Conference on Artificial Intelligence (IJCAI).

[50] Lee J.S., Kim C.R., Lim S.T., Kim Y.-H. (2025). Short-term Efficacy and Safety of Faricimab Switching in Neovascular Age-related Macular Degeneration Previously Treated with Anti-vascular Endothelial Growth Factor. J. Retin..

[51] Song W., Fu C., Zheng Y., Zhang Y., Chen J., Wang P. (2024). Batch Image Encryption Using Cross Image Permutation and Diffusion. J. Inf. Secur. Appl..

