# Supplementary material for: A Rationale-Conditioned Image-Contrast Audit of OCT Dependence in Vision-Language Models for Anti-VEGF Treatment-Response Prediction
Source: Bioengineering (Basel). 2026 Aug 12;13(8):912. doi: 10.3390/bioengineering13080912 (PMC13509487; doi:10.3390/bioengineering13080912)
Supplement: Supplementary file 1 [file bioengineering-13-00912-s001.zip › bioengineering-4464909-supplementary.pdf]

Supplementary Materials

A Rationale-Conditioned Image-Contrast Audit of OCT Dependence in Vision-Language Models for Anti-VEGF Treatment-Response Prediction

Wonbong Jang, Gwon Yul Jo, Siyun Lee, Shin Jeong Yoon, Gi Young Lee, Hee-Eun Lee, Tae Hyung Kim, Jong Won Baek, and Joonhyung Kim

Correspondence: Joonhyung Kim (ophdrjhkim@gmail.com)

S1. Scope of the Supplement

This supplement reports implementation information recoverable from the locked study report and maps the reported protocol labels to current public model repositories. It does not introduce new experiments or results. Generated rationales are treated as observed conditioning variables and are not assumed to be faithful representations of latent computation. Exact historical checkpoint commits, independent weight checksums, and the complete software/hardware environment were not preserved; the public mappings below therefore support model-family identification rather than bit-for-bit reconstruction.

S2. Cohorts, Endpoints, and Data Partitioning

The internal cohort comprised 218 APTOS-2021 eyes with available fluid masks, partitioned at the patient level into 128 training, 21 validation, and 69 test eyes. Bilateral eyes, when present, remained within the same partition. The primary target was the dataset-provided binary continue/stop challenge label; it was not independently re-adjudicated by retina specialists. The secondary responder target was positive when central subfield thickness decreased by at least 25 μm or when the source-defined visual-acuity improvement variable (ΔVA) was at least 0.1; the OCT4DME source records pre- and post-treatment visual acuity in logMAR. Five pre-treatment metadata fields were used: age, sex, planned intravitreal agent, baseline visual acuity, and baseline central subfield thickness. Post-treatment and change variables were excluded from prompts.

The 100-eye second-site cohort was evaluated only as a heterogeneous cross-site distribution-shift stress test. It combined multiple macular and proliferative retinal diagnoses and several intravitreal agents, including two dexamethasone/Ozurdex cases; it is not a harmonized anti-VEGF external-validation cohort.

S3. Public Checkpoint Mapping and Provenance

Table S1. Mapping of protocol labels to public model repositories and current public revisions inspected during manuscript revision.

Protocol label	Public repository / subfolder	Current public revision inspected for documentation	Historical checkpoint status
RetinaVLM-Specialist	RobbieHolland/RetinaVLM / RetinaVLM-Specialist	632c8e25c43f344ab32a60aa1fba3ac64a071593	OCT-specialized research comparator. Exact historical commit and independent weight checksum were not preserved.
LLaVA-Med-v1.5-Mistral-7B	microsoft/llava-med-v1.5-mistral-7b	576a72afe3b11abadadbe17e3519752629d47fed	Biomedical-generalist research comparator. Exact historical commit and independent weight checksum were not preserved.
Qwen3.6-27B	Qwen/Qwen3.6-27B	1b559cf (weight-upload revision)	General-purpose 27B VLM; adapted with 4-bit QLoRA. Exact historical commit and independent weight checksum were not preserved.

The repository mappings identify the public model families corresponding to the protocol labels. The listed revisions are the public revisions inspected for documentation during manuscript revision; they are not asserted to be byte-identical to the historical checkpoints used in the locked execution. Independent historical weight-file checksums were not preserved.

Additional source-code provenance. The archived analysis-code checkout was OCT_LLM_XAI commit 6ce09da9855d1de2def6811491231e7a4d6d4185, with SpecialistVLMs commit 6988375abc61de46aebc279afc16a8642386dda6. These source identifiers document the environment reconstructed during revision and do not establish byte identity of the historical model weights.

S4. Common OCT Rendering and Prompt Schema

Canonical image rendering. The full pre-treatment OCT B-scan panel was retained after a fixed left crop at x=520 pixels and resized to width 720 pixels. The en-face infrared panel and 200-μm scale bar were excluded. Each backbone subsequently applied its native image processor.

Detailed preprocessing. Before each native processor, the OCT panel [x=520, W] was converted to grayscale, contrast-stretched using the 2nd and 98th intensity percentiles, vertically content-fitted while retaining 10% upper and 30% lower margins around the detected retinal content, and Lanczos-resized to a width of 720 pixels. RetinaVLM then used its single-channel 192 x 192 normalization, LLaVA-Med used the checkpoint-specific CLIP image processor, and Qwen used its native AutoProcessor.

Generated-rationale supervision. The semantic target contained four ordered components:

1. Visual findings: presence or absence of intraretinal fluid, subretinal fluid, and pigment epithelial detachment.
2. Pathophysiology: inference of disease activity from the supplied image without explicitly providing the diagnosis.
3. Treatment-related target: the dataset-provided continue or stop label.
4. Predicted response: the composite good- or poor-responder target.

Metadata prefix. For metadata variants, the prompt prepended age, sex, planned intravitreal agent, baseline visual acuity, and baseline central subfield thickness. The exact lexical wrappers followed each backbone's chat template; post-treatment values and outcome changes were never inserted. At test time, each model generated a new rationale from the real OCT and optional pre-treatment metadata; the treatment label and post-treatment outcomes were not included in the prompt.

Verbatim common SFT instruction. This is a pre-treatment macular OCT B-scan. Reason step by step:
Step 1 (Visual findings): state which retinal fluid biomarkers are present — IRF (intraretinal fluid), SRF (subretinal fluid), PED (pigment epithelial detachment).
Step 2 (Pathophysiology): from the image alone, infer the underlying disease activity.
Step 3 (Clinical decision): based on the findings and any provided clinical context, decide per guideline whether to continue or stop anti-VEGF therapy. State exactly 'Decision: continue' or 'Decision: stop'.
Step 4 (Predicted treatment response): predict the composite response to anti-VEGF — anatomic central-subfield-thickness change, visual-acuity change, and fluid resolution — and conclude whether this is a good responder or a poor responder.

Backbone wrappers and decoding. Backbone-native wrappers were retained: the RetinaVLM query interface, the LLaVA-Med mistral_instruct template with an image token, and Qwen apply_chat_template. Free-form generation used greedy decoding (do_sample=False; temperature and top_p sampling were not used).

S5. Adaptation Arms and Hyperparameters

Table S2. Adaptation arms, training settings, and known limitations recoverable from the locked report.

Item	Specification
Arm A	Zero-shot evaluation; no adapter training.
Arm B	Factual four-step generated-rationale supervised fine-tuning with token cross-entropy.
Arm C	Arm B plus L = L_LM + 0.5 × KL(q_fluid || p_attention). Attention rollout used head averaging plus a residual term, adaptive pooling to a 6×6 grid, and a smoothed pooled fluid-mask target. Not available for Qwen.
Arm D	Arm B plus counterfactual fluid-removal rows. Fluid masks were dilated with an elliptical kernel (size 21, one iteration) and inpainted with OpenCV TELEA; the treatment target was flipped to “stop.” This arm is exploratory because the counterfactual rule is not universally clinical.
LoRA	Rank 16; alpha 32; dropout 0.05; no bias; target modules q_proj and v_proj; task type CAUSAL_LM.
Optimization	Three epochs; AdamW; learning rate 1×10−4; gradient-norm clipping at 1.0.
Quantization	Qwen3.6-27B used 4-bit QLoRA.
Training data	Arms B/C used factual training rows only; arm D used factual plus counterfactual rows. Adapter optimization used the 128 training eyes; the validation set was used for checkpoint selection; test eyes were excluded.
Randomness	One training realization per cell. Split seed=0 and bootstrap seed=0. Repeated-seed training was not performed. Training used a fixed seed of 42 for sample-order shuffling.

S6. Rationale-Conditioned Direct-Contrast Scoring

Let V_real denote the clinical OCT, V_grey the uniform-grey input, M_real the rationale generated from V_real, and Y(V,M) the terminal probability assigned to "continue." The primary quantity was D_direct = Y(V_real,M_real) − Y(V_grey,M_real). Because M_real was fixed, this quantity does not measure the mediated pathway V → M(V) → Y. M_real was the model's own test-time output rather than text copied from the supervised target or constructed from the test label.

The exact hard-break suffix was:

=== CLINICAL DECISION === Options: [continue, stop] Final Choice:

Option-order symmetry was implemented by scoring both [continue, stop] and [stop, continue] orders and averaging. Length-normalized continuation log probabilities were converted to a two-option probability. Each eye required eight scoring passes: two answer orders × two image conditions × two option phrases. The legacy code variable pmiAUC denotes the AUC of this uniform-grey single-null contrast; it is not formal pointwise mutual information.

Exact scored continuations. The continuation strings were exactly " continue" and " stop", including the leading space. The second suffix reversed only the displayed option list to Options: [stop, continue]. The terminal audit used continuation log probabilities and did not sample a decoded answer.

Table S3. Prompt, conditioning, and direct-contrast scoring schema.

Component	Specification
Canonical image input	Full pre-treatment OCT B-scan after fixed left crop beginning at x=520 pixels and resizing to width 720 pixels; each backbone then applied its native processor.
Generated-rationale target	Four ordered components: IRF/SRF/PED findings; inferred disease activity; dataset-provided continue/stop target; composite responder target.
Metadata prefix	Age, sex, planned intravitreal agent, baseline visual acuity, and baseline central subfield thickness. Post-treatment values and outcome changes were excluded.
Hard-break suffix	=== CLINICAL DECISION === Options: [continue, stop] Final Choice:
Option-order control	Both [continue, stop] and [stop, continue] orders were scored and averaged.
Probability readout	Length-normalized continuation log probabilities were converted to a two-option probability.
Scoring passes	Eight passes per eye: two answer orders × two image conditions × two option phrases.
Legacy variable	pmiAUC is the historical code label for the uniform-grey single-null contrast AUC; it is not formal pointwise mutual information.
Rationale interpretation	The generated rationale is an observed conditioning variable and was not validated as a faithful representation of latent computation.

S7. Fluid Masks and Exploratory Grounding Interventions

Fluid masks were produced by a fixed ReLayNet-based segmenter fine-tuned on Duke/UMN OCT data. The class-9 fluid channel was mapped to the canonical B-scan. The masks cover fluid compartments but do not represent PED. No APTOS pixel-level reference standard or independent retina-grader validation was available. Accordingly, attention-KL and synthetic occlusion analyses are exploratory and do not establish faithful localization or causal treatment use.

S8. Statistical Analysis and Output Coverage

AUCs were summarized with 5000-replicate patient-clustered bootstrap 95% confidence intervals using seed 0. Confidence intervals were nominal. Bootstrap p-values relative to AUC 0.5 were adjusted with the Benjamini–Hochberg procedure within the 22-cell decision family and separately within the 22-cell responder family. No equivalence margin was prespecified. A confidence interval including 0.5 was therefore considered inconclusive rather than evidence of equivalence. LLaVA-Med newline-degenerate generations were excluded from complete-case AUCs and reported through output coverage.

S9. Reproducibility and Security Scope

Table S4. Reproducibility and security information supplied and known limitations.

Domain	Information supplied	Known limitation
Checkpoint identity	Protocol-label-to-public-repository mappings and current public revisions inspected for documentation are listed in Table S1.	The original historical commits and independent weight-file checksums were not preserved; bit-for-bit checkpoint identity is not claimed.
Prompting and training	Target schema, metadata fields, hard-break suffix, LoRA settings, and optimization parameters are reported.	Backbone-specific lexical wrappers follow native chat templates; exact historical package build numbers were not retained.
Hardware/software	The pipeline used Python, PyTorch, Hugging Face Transformers/PEFT, bitsandbytes for 4-bit QLoRA, and OpenCV for inpainting.	Exact package builds, CUDA version, GPU model, and runtime were not preserved in the locked report and are not retrospectively inferred.
Data security	Both cohorts were de-identified; second-site access was institutionally governed.	Encryption methods were discussed but not experimentally evaluated.

Medical imaging pipelines should combine de-identification with encryption at rest and in transit, role-based access control, audit logging, and governed data access. Batch-image encryption methods such as the cross-image permutation and bidirectional diffusion approach of Song et al. may provide additional safeguards, but preservation of diagnostically relevant OCT features must be validated before clinical use.

Supplementary Reference

Song, W.; Fu, C.; Zheng, Y.; Zhang, Y.; Chen, J.; Wang, P. Batch Image Encryption Using Cross Image Permutation and Diffusion. Journal of Information Security and Applications 2024, 80, 103686. https://doi.org/10.1016/j.jisa.2023.103686.
